# Liver and intestinal protective effects of *Castanea sativa* Mill. bark extract in high-fat diet rats

**DOI:** 10.1371/journal.pone.0201540

**Published:** 2018-08-06

**Authors:** Roberta Budriesi, Fabio Vivarelli, Donatella Canistro, Rita Aldini, Clara Babot Marquillas, Ivan Corazza, Romana Fato, Silvia Cirillo, Christian Bergamini, Antonia D’Errico, Cristiano Bolchi, Monica Cevenini, Alessio Degiovanni, Maria Frosini, Luca Camarda, Alberto Chiarini, Matteo Micucci

**Affiliations:** 1 Department of Pharmacy and Biotechnology, Alma Mater Studiorum-University of Bologna, Bologna, Italy; 2 Department of Specialistic, Experimental and Diagnostic Medicine, Alma Mater Studiorum-University of Bologna, S. Orsola Hospital, Bologna, Italy; 3 "F. Addari" Institute of Oncology and Transplant Pathology, Department of Specialistic, Diagnostic and Experimental Medicine Department, Alma Mater Studiorum-University of Bologna, Bologna, Italy; 4 Department of Pharmaceutical Sciences “Pietro Pratesi”, Università degli Studi di Milano, Milano, Italy; 5 Department of Surgical and Medical Sciences, Alma Mater Studiorum-University of Bologna, Bologna, Italy; 6 Department of Life Sciences, University of Siena, Siena, Italy; 7 GVM Care & Research, Lugo, Ravenna, Italy; Universidade do Porto, Faculdade de Farmácia, PORTUGAL

## Abstract

The effects of *Castanea sativa* Mill. have been studied in high fat diet (HFD) overweight rats. Natural Extract of Chestnut bark (*Castanea sativa* Mill.) (ENC^®^), rich in ellagitannins, has been studied in 120 male Sprague-Dawley rats, divided in four groups. Two groups were controls: regular (RD) and HDF diet. Two groups received ENC^®^ (20 mg/kg/day): RD + ENC^®^ and HFD + ENC^®^. At baseline and at 7, 14 and 21 days, weight gain, serum lipids, plasma cytokines, liver histology, microsomial enzymes and oxidation, intestinal oxidative stress and contractility were studied. HFD increased body weight, increased pro-inflammatory cytokines, induced hepatocytes microvescicular steatosis, altered microsomial, increased liver and intestinal oxidative stress, deranged intestinal contractility. In HFD-fed rats, ENC^®^ exerted antiadipose and antioxidative activities and normalized intestinal contractility, suggesting a potential approach to overweight management associated diseases.

## Introduction

Unhealthy sedentary lifestyle and excessive eating have influenced the increase of closely related disturbances, such as obesity, insulin resistance, hypertension, and dyslipidemia, and the outstanding example of metabolic syndrome [1]. Metabolic syndrome is characterized by chronic dysregulation of metabolic and immune processes [2], low-grade inflammation [3], antioxidant impairment [4], xenobiotic metabolism homeostasis disruption [5], vascular smooth muscle contractility alterations [6]. Increased oxidative stress (OS) in obese patients [7] appears to be involved in the onset and progression of the pathological alterations of different organs [8–12]. Obesity-induced inflammatory dysfunctions may be, at least in part, reversible by “Comprehensive lifestyle interventions” [13]. Nutraceuticals use in the preclinical phase is a “proactive reverse approach” tool to health conditions in prevention time [14] and studies are being conducted to understand the mechanisms of action of bioactive nutraceutical compounds.

Traditionally, *Castanea sativa* Mill. leaves or bark has been widely used against various diseases, for its high antioxidant [15], cardioprotective activity [16], antihelmintic, antibacterial and antiviral effects [17], neuroprotective activity [18, 19], antispasmodic action on gallbladder [20] and intestine [15, 21].

High levels of tannins and their antioxidant activity have suggested that *Castanea sativa* Mill. bark extract associated to diet as a supplement, can help to prevent and treat the pathological processes underlying obesity.

In this study, we show the protective effect of Natural Extract of Chestnut [ENC^®^ (*Castanea sativa* Mill. bark extract)] on body weight gain, liver inflammation and function, liver and intestinal oxidative state, gastric and intestinal contractility in high-fat diet rats in order to propose its use in the prevention and possibly co-management of obesity.

## Materials and methods

### Plants materials and chemicals

Bark extract of a *Castanea sativa* Mill. (ENC^®^), (supplied by SilvaTeam S.p.a., San Michele Mondovì, Italy) was obtained with a process previously described [21]. The powder (92–95% dry matter) is made by 77% tannins and its chemical composition has been previously characterized by HPLC-DAD-MS analysis. [16]. It is rich in phenolic compounds such as: castalin, vescalin, castalgin, vescalgin, ellagic acid and gallic acid. The amount of ellagic acid and of gallic acid found in ENC^®^ were 10.69 ± 0.28% and 24.01 ± 0.57% respectively. The extract used in the study has been obtained as previously described and the percentage composition of the phenolic compounds has been obtained. Further information about ENC^®^ is available at SILVATEAM website (http://it.silvateam.com/). Detailed information about chemicals are listed in S1 File.

### Animals and experimental design

**Fig 1:**
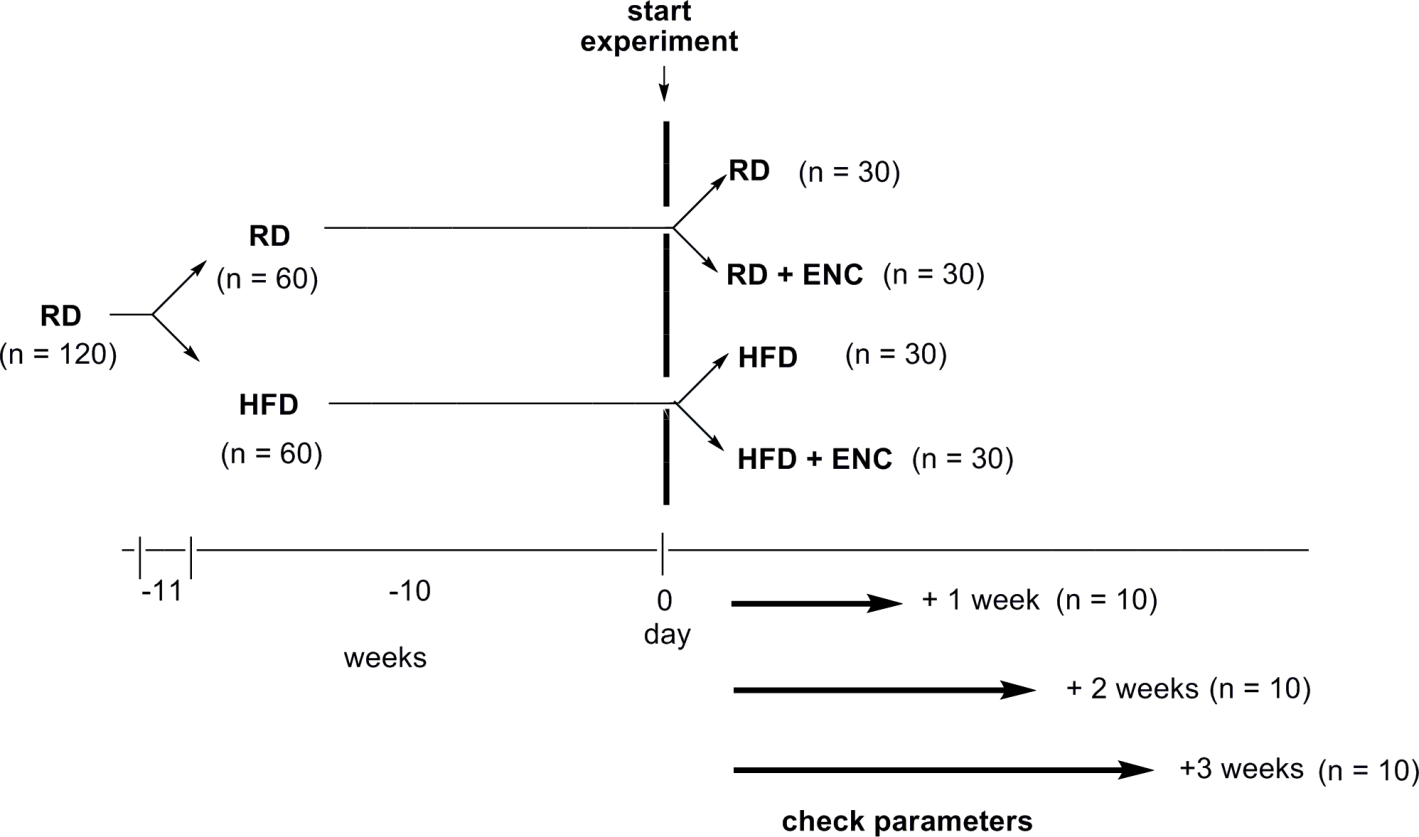
Overview of the experimental design. After one-week housing period, animals were randomly assigned to RD or HFD groups (10-weeks fattening period). HFD and control rats were further split into 4 groups as depicted. Animals were treated for 7, 14 or 21 days during which they received ENC^®^ (20 mg/kg/day) or vehicle (tap water).

120 male Sprague-Dawley rats were purchased from Envigo RMS S.R.L. San Pietro al Natisone (Udine, Italy) at 9 weeks of age weighing 270–300 g. They were housed over 7 days adaptation under 12h-light/12h-dark cycle, 22°C, 60% humidity, with water and food ad *libitum*. The rats were split randomly into two groups and fed specific diets as follows: regular diet (RD, n = 60: 18.7% crude protein, 5.6% crude fat, 4.5% crude fibre by Mucedola s.r.l.); high fat diet (HFD, n = 60: 23% crude protein, 34% crude fat, 5% crude fibre, by Mucedola s.r.l.). HFD has been reported in S2 File. Animals maintained the dietary regimens described for 10 weeks, then each group was assigned to the treatment as follows: RD animals with vehicle (tap water) (n = 30), RD animals with ENC^®^ 20 mg/kg administered by gavage (n = 30), HDF group with vehicle (tap water) (n = 30), HFD diet with ENC^®^ 20 mg/kg administered by gavage (n = 30). (Fig1). The dose of ENC^®^ to be administered to rats was extrapolated by using the translational dose calculation [22], which express the human equivalent dose (HED, mg/kg) as:

$$HED=Animal\;dose\times \frac{Animal\;Km}{Human\;Km}$$

where *K*_m_ was 6 and 37 for rat and human, respectively. As Abidov used 300 mg/day in man, a value in agreement with the human dose of 225 mg/day [23], the resulting final dose for rat was thus calculated to be 20 mg/kg. Furthermore, 21 days of treatment was chosen as it is the most common duration period to highlight metabolic changes and possible toxic effects and to better evaluate the time-course of the observed changes. At every end point (7, 14, 21 days) the animals were sacrificed by decapitation (n = 10/ end point) and the parameters reported.

### Ethical statements

The work was conducted according to the guidelines set forth to EU Directive 2010/63/EU and to ARRIVE guidelines [24]. The protocol was approved by the Institutional Ethics Committee of the University of Bologna (Protocol 21/79/14) and transmitted to the Ministry of Health. Details of Humane end points.docx are in S3 File.

### Body-weight gain and food intake

Body weight and food consumption measurements started the first day of the study and continued for the entire experiment (3 weeks).

### Determination of lipids, transaminases and inflammation

#### Serum lipids and transaminases

Triglyceride, HDL-cholesterol, LDL-cholesterol, total cholesterol and transaminases (ALT and AST) concentrations were measured by using commercially available kits (see S4 File).

#### Plasma inflammatory response

Biomarkers of inflammation have been quantitatively evaluated in RD- and HFD- rats, in basal conditions and after ENC^®^ addition: IL-1α, IL-1β, IL-2, IL-5, IL-6, IL-7, IL-12p70, IL-17A, IL-18, and TNF-α (pro-inflammatory) and IL-4, IL-10, IL-13 (anti-inflammatory), by the Luminex MAGPIX^®^ system (Millipore Corp., Billerica, MA, USA). For details, see S4 File).

### Histology

Liver necro-inflammatory score and architectural changes, fibrosis and cirrhosis were assessed after haematoxylin and eosin tissue staining (See S4 File).

### Hepatic Phase I, Phase II and antioxidant enzyme activities

Tissue collection and preparation of hepatic subcellular fractions, Phase I and II, antioxidant enzymes and their activities, listed below, were assessed as reported in S4 File).

#### Phase I

NADPH-(CYP)-c-reductase (CYP-red), Aminopyrine N-demethylase (APND)- CYP3A1/2, p-Nitrophenol hydroxylase (p-NFH)-CYP2E1, Pentoxyresorufin *O*-dealkylase (PROD)-CYP2B1/2, Ethoxyresorufin *O*-deethylase, (EROD)-CYP1A1, Methoxyresorufin *O*-demethylase (MROD)-CYP1A2, Ethoxycoumarin *O*-deethylase (ECOD)-CYP1A1/2.

#### Phase II

Glutathione S-transferase (GST) activity, UDP-glucuronosyl transferase (UDPGT).

#### Antioxidant enzymes

Catalase (CAT) activity, NAD(P)H:quinone reductase (NQO1), Oxidised glutathione reductase activity (GSSG-red), Superoxide dismutase activity (SOD).

### Oxidative stress evaluation in ileal and colonic tissues

OS biomarkers were evaluated in colon and ileum by assay: Details are reported in S4 File).

### Spontaneous and induced contractility studies

Gastric fundus basal contractility was investigated. Ileum and proximal colon induced contractility were studied against cholinergic receptors and calcium channels (S4 File).

### Data presentation and statistical analysis

Results are presented as mean ± standard deviation (S.D.) or error standard (S.E.M.) from n rats. The experimental design was subjected to ‘*a priori* power analysis’ to ensure that the size of treated- and control- groups was adequate (G*Power v. 3.1.9.2). To improve clarity, since the content of serum lipids and transaminase, plasma pro- anti-inflammatory cytokines, MDA in ileum and colon as well as hepatic Phase I, II and antioxidant enzyme activities did not change significantly between day 0 and day 21 in both RD- and HFD-rats, these have been pooled and reported as RD 0–21 or HFD 0–21. Plasma cytokine content was reported as percent changes of the baseline value [100 (%)] of RD-rats. Data were compared by ANOVA followed by Bonferroni’s post hoc tests and in all comparisons, P value less than 0.05 was considered significant. Before performing the analysis, ANOVA assumption were always tested using the method of Bartlett (data sampled from populations with identical SDs) and Kolmogorov and Smirnov (data sampled from populations that follow Gaussian distributions).

Changes in spontaneous contractions of gastric fundus were considered significant when they were higher than 10% variations in each range [25].

Functional induced contractility of agonist Carbacol (CCh) was expressed as pEC_50_ values. The antagonism activity of atropine against CCh was expressed as pA_2_ values determined from Schild plots [26] constrained to slope –1.0, as required by theory [27]. Differences between mean values were performed by using a two-tailed unpaired Student’s *t*-test for continuous data. In all comparisons, *P* value less than 0.05 was considered significant.

## Results

### Body weight gain and food intake

**Fig 2:**
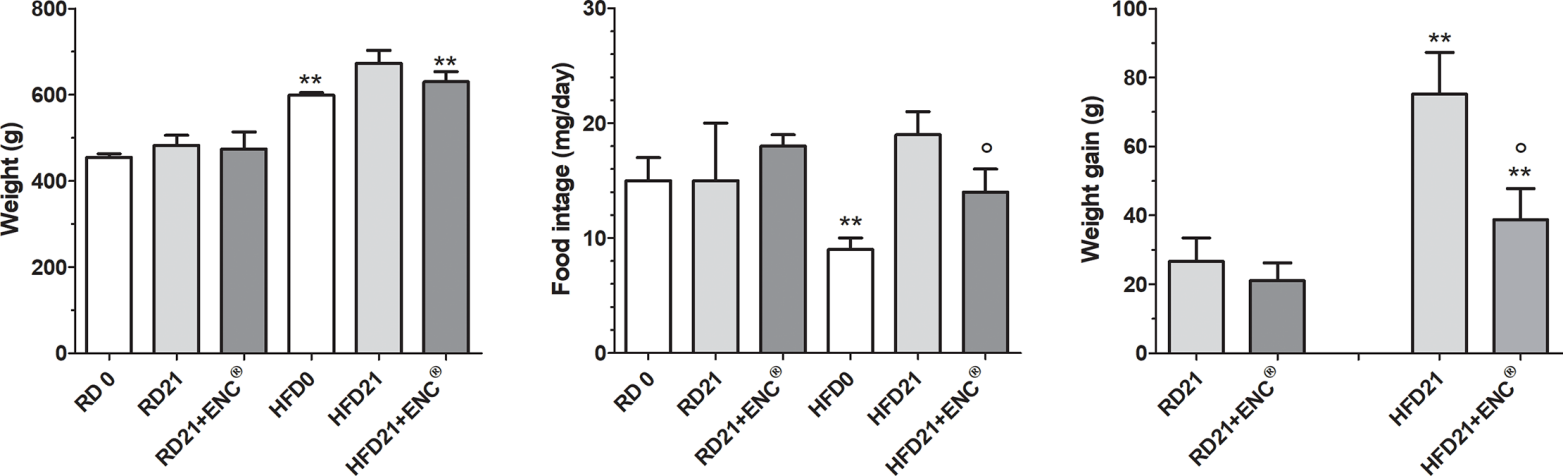
Body weight, food intake and weight gain of RD-rats, HFD-rats with or without ENC^®^ (20 mg/kg/day) for 21 days. RD 0 or HFD 0: data measured at day 0; RD 21 or HFD 21: data measured at day 21. Results are reported as mean ± S.D. (n = 10/group) and analyzed by using ANOVA followed by Bonferroni post test. **P < 0.01 vs RD-fed group, same time. °*P* < 0.05 vs HFD21.

Three weeks of HFD increased significantly rat body weight, with respect to RD (598.1 ± 41.4 g *vs* 455.8 ± 20.0 g, *P* < 0.01), despite a general lower food intake of the former group (Fig 2). In RD-rats, weight, weight gain and food intake did not show significant differences with ENC^®^, while in HFD-rats ENC^®^ caused a significant decrease in food intake and weight gain with respect to HFD-rats (Fig 2).

It is worth outlining that 21 days of ENC^®^ treatment did not cause any death in either RD- or HFD-rats. Moreover, physical observations of treated animals showed no signs of changes in the skin, fur, eyes mucous membrane, behavior patterns, tremors, salivation, suggesting an apparent safe profile of ENC^®^ treatment.

### Liver gross appearance and histology

**Fig 3:**
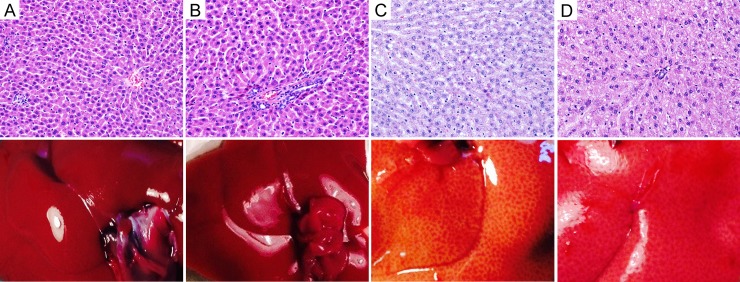
**Histology of liver tissues (upper panels) and gross appearance of the liver (lower panels) of RD-rats, HFD-rats with or without ENC**^**®**^ **(20 mg/kg/day) for three weeks**. A: RD control rat: normal rat liver showing central vein and a regular parenchyma. B: RD rat after ENC^®^: the liver shows a normal portal tract and mild hepatocyte pleomorphism. C: HFD rat: the hepatocytes show a moderate microvescicular steatosis. D: HFD rat after ENC^®^: microvescicular steatosis is evident only in scattered hepatocytes. (H&E) 10x.

RD-rats presented normal liver histology, while HFD induced a moderate hepatocytes microvesicular steatosis; after ENC^®^ microvesicular steatosis was only in scattered hepatocytes (Fig 3), with a regression of liver steatosis. The livers did not present any sign of necroinflammatory score (arbitrary score of 0 on a scale range from 0 to 18). Similarly, the gross appearance of the liver showed an enlargement and lightening in color that decreased after ENC^®^ assumption. Histological parameters are detailed in S1 Table.

### Lipids, transaminases and inflammation

#### Serum lipids and transaminases

As reported in Table 1, HFD-rats serum cholesterol levels were higher than those of RD-rats (*P* < 0.01 *vs* RD 0–21). Cholesterol was significantly lowered by ENC^®^ administration in HFD-rats (-10%, *P* < 0.05). LDL Cholesterol was higher in HFD rats than in controls (*P* < 0.05 *vs* RD 0–21); ENC^®^ decreased LDL levels in HFD-rats (*P* < 0.05 *vs* HFD 0–21), while it left them unchanged in RD-rats. At the beginning of the study, HDL Cholesterol was lower in HFD- than in RD-rats (*P* < 0.05 *vs* RD 0–21). ENC^®^ increased HDL in the HFD group (*P* < 0.05 *vs* HFD0-21), whereas it did not affect that of RD-rats. Triglycerides were increased by about 63% in HFD with respect to RD- rats (*P* < 0.01 *vs* RD 0–21); interestingly, in the former group ENC^®^ decreased triglycerides levels (*P* < 0.01 *vs* HFD 0–21). Finally, aspartate transaminases and alanine transaminases were not affected by the treatment in both RD- and HFD-rats. RD-rats and HFD-rats with or without ENC® (21 days).

**Table 1:** Serum parameters of RD-rats and HFD-rats supplemented with or without ENC^®^ for 21 days.

	RD	RD+ ENC^®^^a^	HFD	HFD+ ENC^®^^a^
**Day**	**0–21**	**21**	**0–21**	**21**
**AST**^**b**^	242 ± 60	236 ± 70	237 ± 60	238 ± 60
**ALT**^**c**^	67 ± 23	64 ± 25	60 ± 23	60 ± 21
**Cholesterol**^**d**^	3.16 ± 0.23	3.10 ± 0.19	3.68 ± 0.17**	3.28 ± 0.16°
**LDL Cholesterol**^**d**^	0.90 ± 0.03	0.88 ± 0.09	1.02 ± 0.02*	0.92 ± 0.07°
**HDL Cholesterol**^**d**^	2.34 ± 0.08	2.36 ± 0.03	2.18 ± 0.04*	2.26 ± 0.03°
**Triglyceride**^**d**^	1.24 ± 0.14	1.22 ± 0.16	2.03 ± 0.10**	1.46 ± 0.07°°

a) ENC^**®**^ 20 mg/kg/day.

b) AST, Aspartate transaminases (Units/ml).

c) ALT, Alanine transaminases (Units/ml).

d) Expressed as mmol/l.

To improve clarity, since values between RD 0 day and RD 21 days did not change significantly, they have been pooled; the same was for HFD 0 day and HFD 21 days. Results are reported as mean ± S.D. (n = 10/group) and analyzed by using ANOVA followed by Bonferroni post test.

*P < 0.05

**P < 0.01, *vs* RD-rats, same time.

°*P* < 0.05

°°*P* < 0.01 *vs* HFD 0–21.

#### Inflammatory response

**Fig 4:**
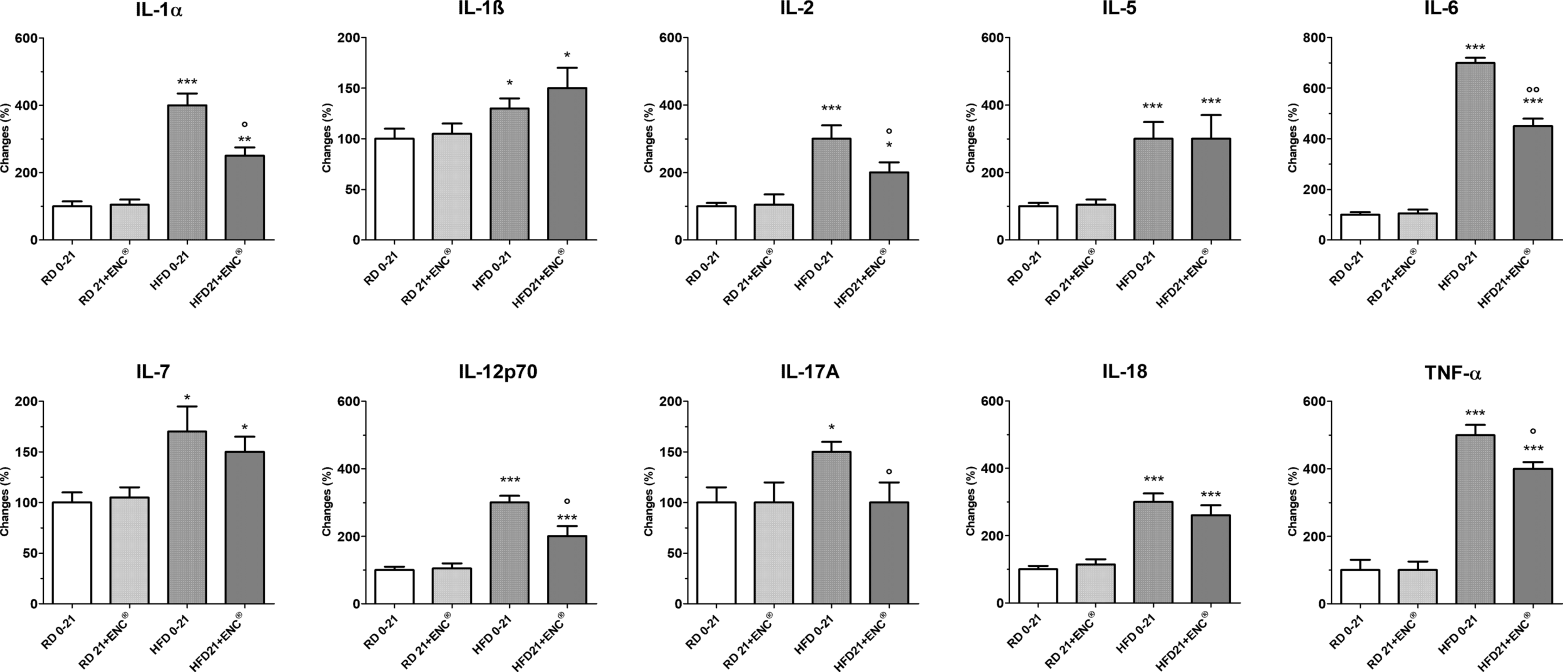
Plasma pro-inflammatory cytokines in RD-rats, and HFD-rats with or without ENC^®^ (20 mg/kg/day) for 21 days. RD0-21 or HFD0-21: pooled data between days 0–21. Data are reported as mean ± S. D. and expressed as a percent changes of the baseline value [100 (%)] of RD-fed rats. *P < 0.05, **P < 0.01, ***P < 0.001 *vs* RD same time. °*P* < 0.05, °°*P* < 0.01 *vs* HFD 0–21 (ANOVA followed by Bonferroni post test).

**Fig 5:**
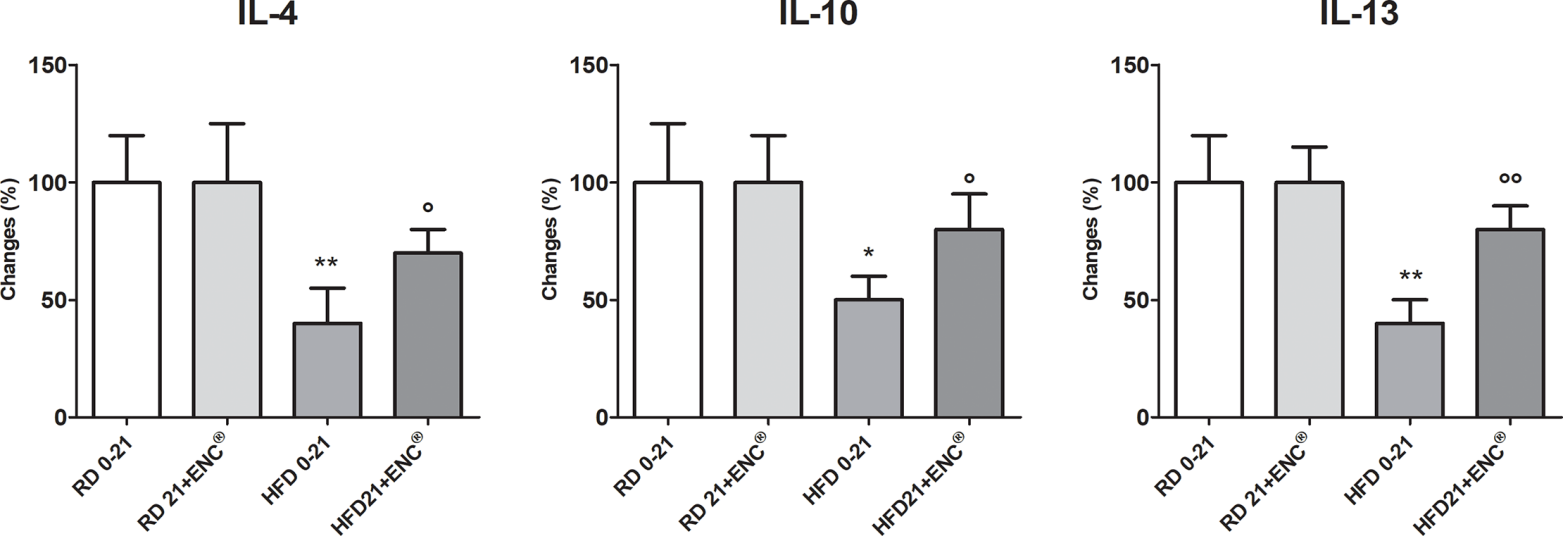
Plasma anti-inflammatory cytokines in RD-rats, and HFD-rats with or without ENC^®^ (20 mg/kg/day) for 21 days. RD0-21 or HFD0-21: pooled data between days 0–21. Data are reported as mean ± S.D. and expressed as percent changes of the baseline value [100 (%)] of RD-rats. *P < 0.05, **P < 0.01, ***P < 0.01 *vs* RD same time. °*P* < 0.05, °°*P* < 0.01 *vs* HFD 0–21 (ANOVA followed by Bonferroni post test).

HFD-rats had higher pro-inflammatory cytokines plasma levels than RD-rats (Fig 4), changing from + ~ 30% (IL-1ß) to +~ 600% (IL-6). In the former group, however, 21 days ENC^®^ decreased significantly IL1α, IL-2, IL-6, IL-12p70, IL17A and TNF-α plasma levels, while it left unchanged those of IL-1ß, IL-5 and IL-18. Interestingly, the anti-inflammatory cytokines IL-4, IL-10 and IL-13 were significantly lower in overweight animals (- 50–60%, *P*<0.05 or 0.01 *vs* RD 0–21) (Fig 5). ENC^®^ reverted this effect, restoring anti-inflammatory cytokines plasma content to values very close to RD-rats. Finally, ENC^®^ did not affect cytokines plasma contents in RD-rats.

### Hepatic Phase I, Phase II and antioxidant enzyme activities

#### Hepatic Phase-I enzymes

**Fig 6:**
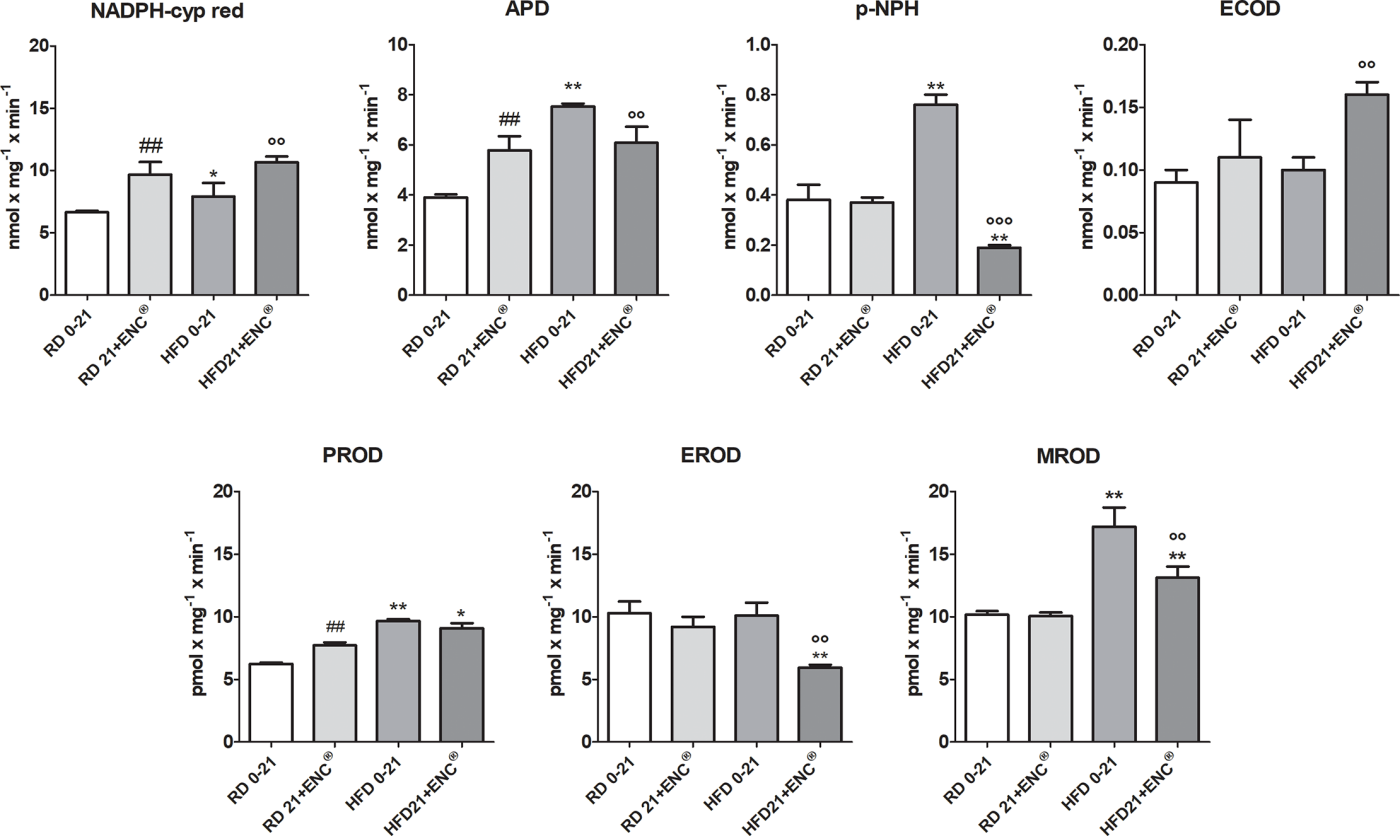
Changes in liver phase-I enzymes in RD-rats, and HFD-rats with or without ENC^®^ (20 mg/kg/day) for 21 days. RD0-21 or HFD0-21: pooled data between days 0–21. Bars represent the means ± S.D. *P < 0.05, **P < 0.01, *vs* RD same time; °*P* < 0.05, °°*P* < 0.01, °°°*P* < 0.001 *vs* HFD0-21; ## *P* <001 vs RD0-21.

In RD-rats, ENC increased the activity NADPH cyp red, APD and PROD, leaving unaffected that of pNPH, ECOD, EROD and MROD (Fig 6). Also HFD-rats reported a widespread increment of microsomal hepatic mono-oxygenases respect to RD-rats. In particular, CYP3A1/2, CYP2E1 and CYP1A2 isoform-linked activities, here measured as APD, p-NPH and MROD, respectively as well as NADPH cyp-red were significantly raised (*P* < 0.01 vs RD0-21) (Fig 6), while ECOD and EROS were not changed. ENC^®^ was effective in counteracting the changes caused by HFD, reducing almost all enzymes increased activities (*P* < 0.01 *vs* HFD 0–21). Hepatic phase-I enzymes full changes are detailed in S2 Table.

#### Hepatic phase-II enzymes

**Fig 7:**
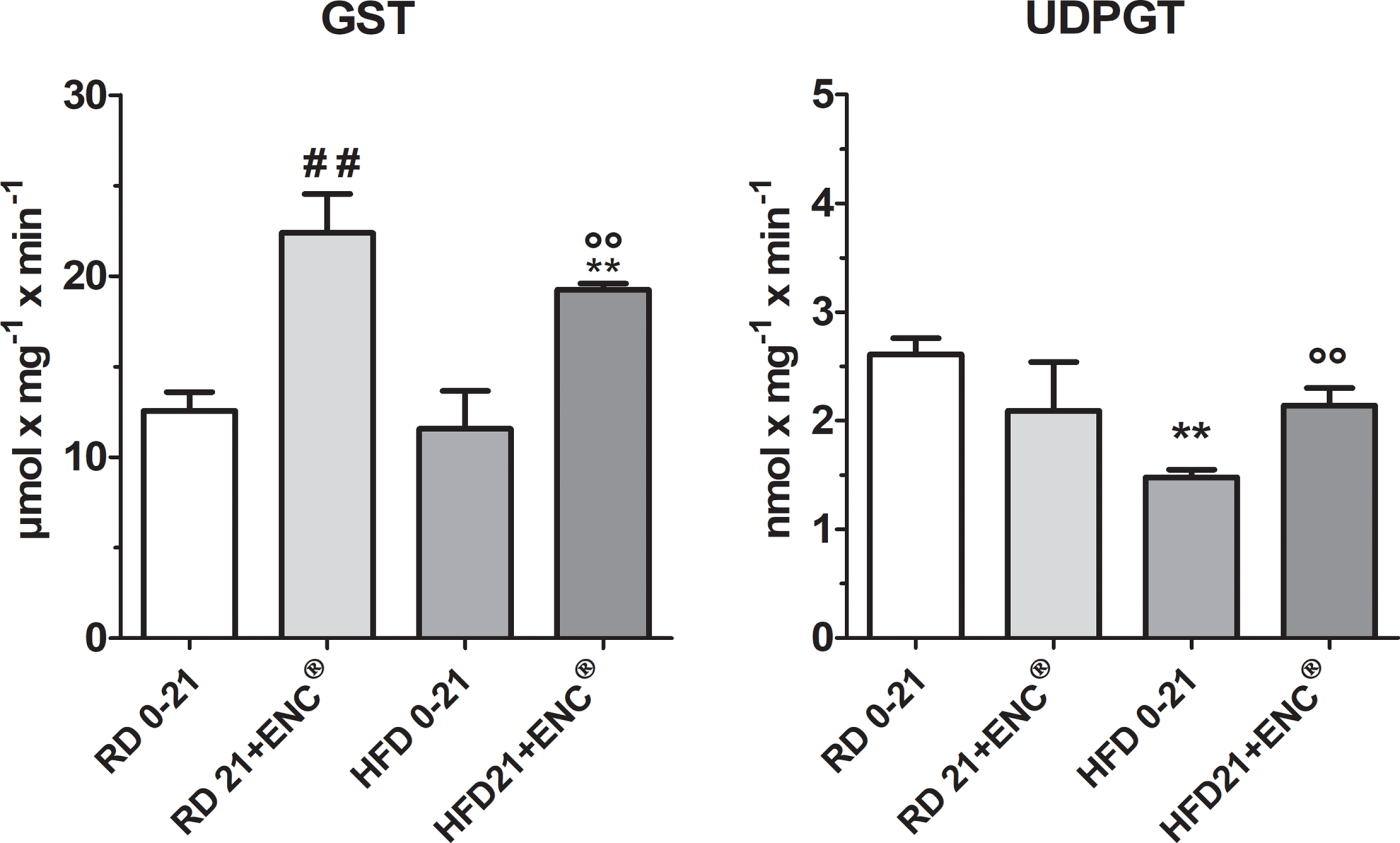
Changes in liver phase-II enzymes in RD-rats, and HFD-rats with or without ENC^®^ (20 mg/kg/day) for 21 days. RD0-21 or HFD0-21: pooled data between days 0–21. Bars represent the means ± S.D. ^##^*P* < 0.01 *vs* RD0-21; **P < 0.01 *vs* RD same time; °°*P* < 0.01 *vs* HFD0-21.

ENC^®^ supplementation induced a significant up-regulation of GST activity in both RD- (≈ 78%, *P* < 0.01) and HFD-rats (≈66%, *P* < 0.01) (Fig 7). In HFD-rats, UDPGT was decreased with respect to RD-rats and ENC^®^ restored the values toward those observed in RD-rats. Detailed information is in S2 Table.

#### Hepatic antioxidant enzymes

**Fig 8:**
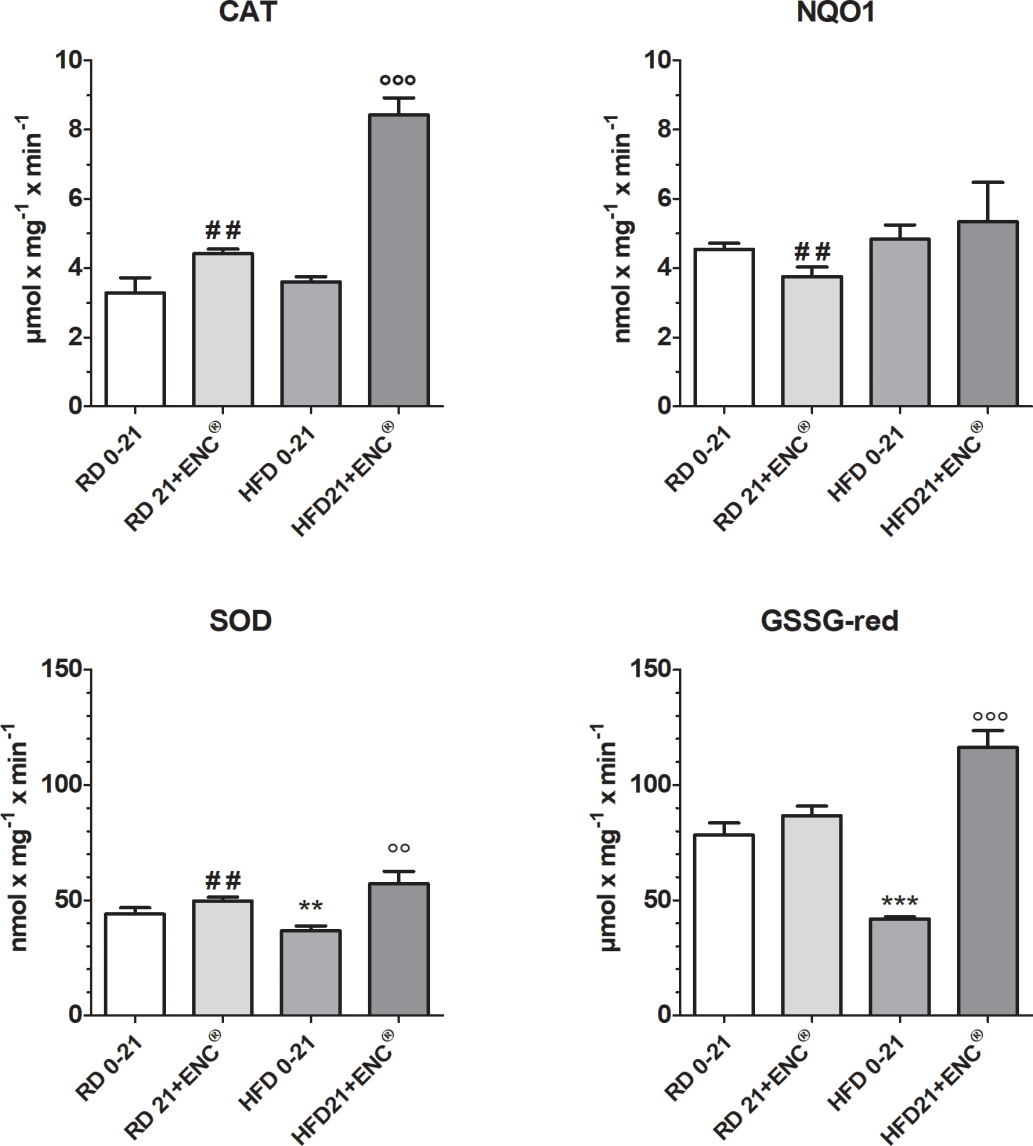
Changes in hepatic antioxidant enzymes after 21 days ENC^®^ supplementation (20 mg/kg/day) in RD-fed and HFD-fed rats. RD0-21 or HFD0-21: pooled data between days 0–21. Bars represent the means ± S.D. of ten measurements performed on ten rats. ** *P*< 0.01 *vs* RD-fed group, same time. °°*P* < 0.01, °°° *P* <0.001 *vs* HFD0-21, ^##^*P* < 0.01 *vs* RD0-21.

Hepatic antioxidant enzymes CAT and NQ01 were similar in RD and HFD rats. On the contrary, as shown in Fig 8, HFD decreased SOD and GSSG-red with respect to RD. In RD feed rats, the treatment with ENC^®^ increased CAT and SOD and decreased NQ01 activities, while in HFD animals, it caused an increment of CAT, SOD and GSSG-red, completely restoring these enzyme antioxidant potential which exceeded the values recorded in RD group (Fig 8). For detailed information about the changes in the time course of antioxidant enzymes activities, see S2 Table.

### Oxidative stress in ileal and colonic tissues

**Fig 9:**
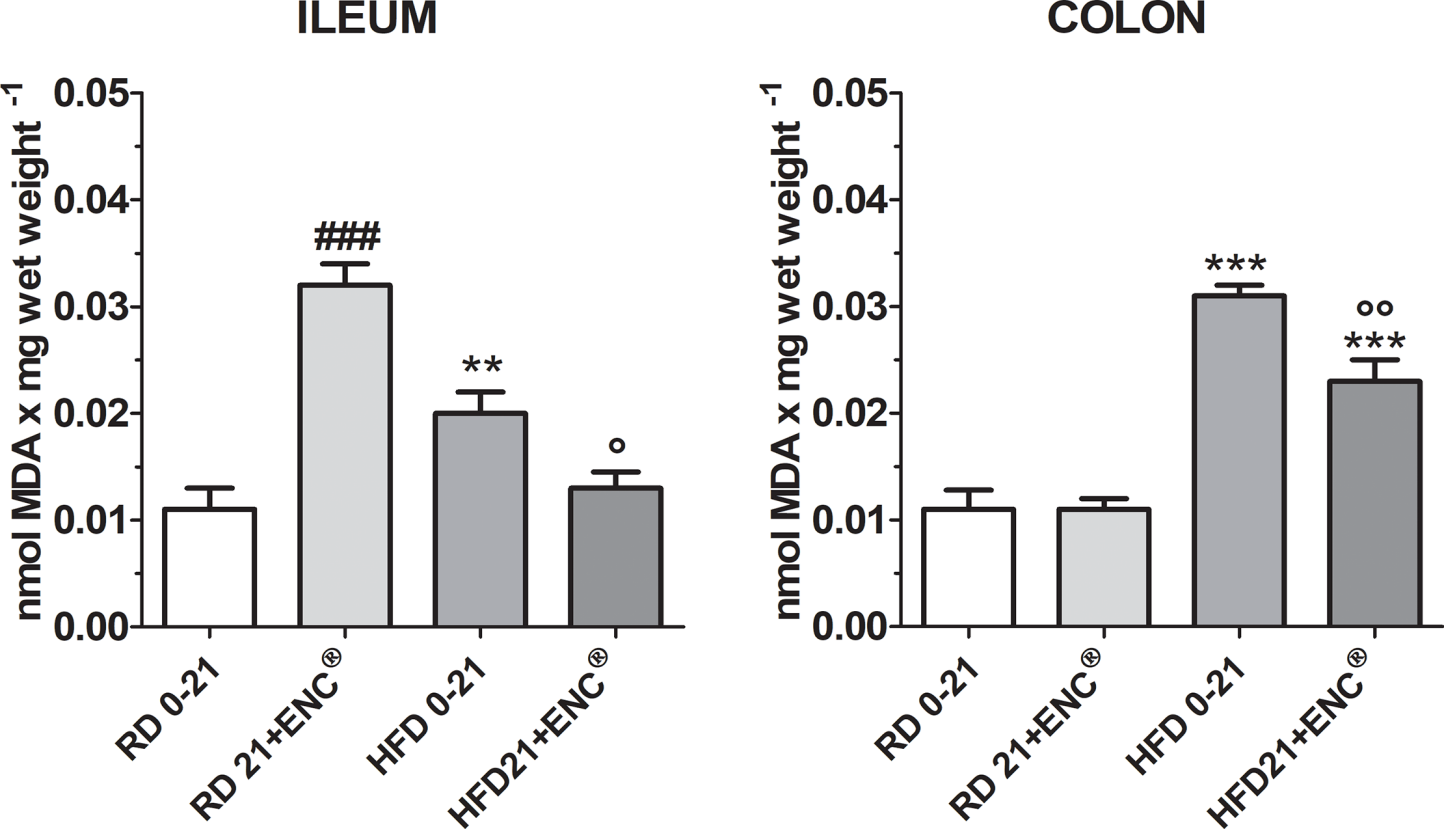
MDA equivalents in ileum and colon homogenates in RD-rats and HFD-rats supplemented with or without ENC^®^ (20 mg/kg/day) for 21 days. Data are expressed as mean ± SD. **P < 0.01, ***P < .001 *vs* RD same time. °*P* < 0.05, °°*P* < 0.01 *vs* HFD0-21 (ANOVA followed by Bonferroni post test).

Malondialdehyde (MDA) assay, a product generated from lipid oxidation, was used to measure the level of OS in the different tissues. HFD-rats showed a significant increase in the MDA values respect to RD-rats (*P* < 0.01 in the ileum and *P*<0.001 in the colon) confirming that a HFD induces OS (Fig 9). Interestingly, the RD-rats, treated with ENC^®^ for 21 days had a high MDA value in the ileum suggesting that the phenolic compounds present in the ENC^®^ may have a pro-oxidant effect on this tissue at the dose of extract used. Nevertheless, 21 days of ENC^®^ administration was able to counteract HFD-induced OS in the ileum (Fig 9). The pro-oxidant effect of ENC^®^ in RD-rats was not evident in colon tissue. On the other hand, OS induced by the HFD on this tissue was 2.5 times higher than that of the RD-rats (*P* < 0.001); after 21 days ENC^®^treatment MDA was significantly reduced (*P* < 0.01 *vs* HFD 0–21) confirming the antioxidant properties of ENC^®^ (Fig 9).

### Spontaneous and induced contractility studies

#### Spontaneous contractility

**Fig 10:**
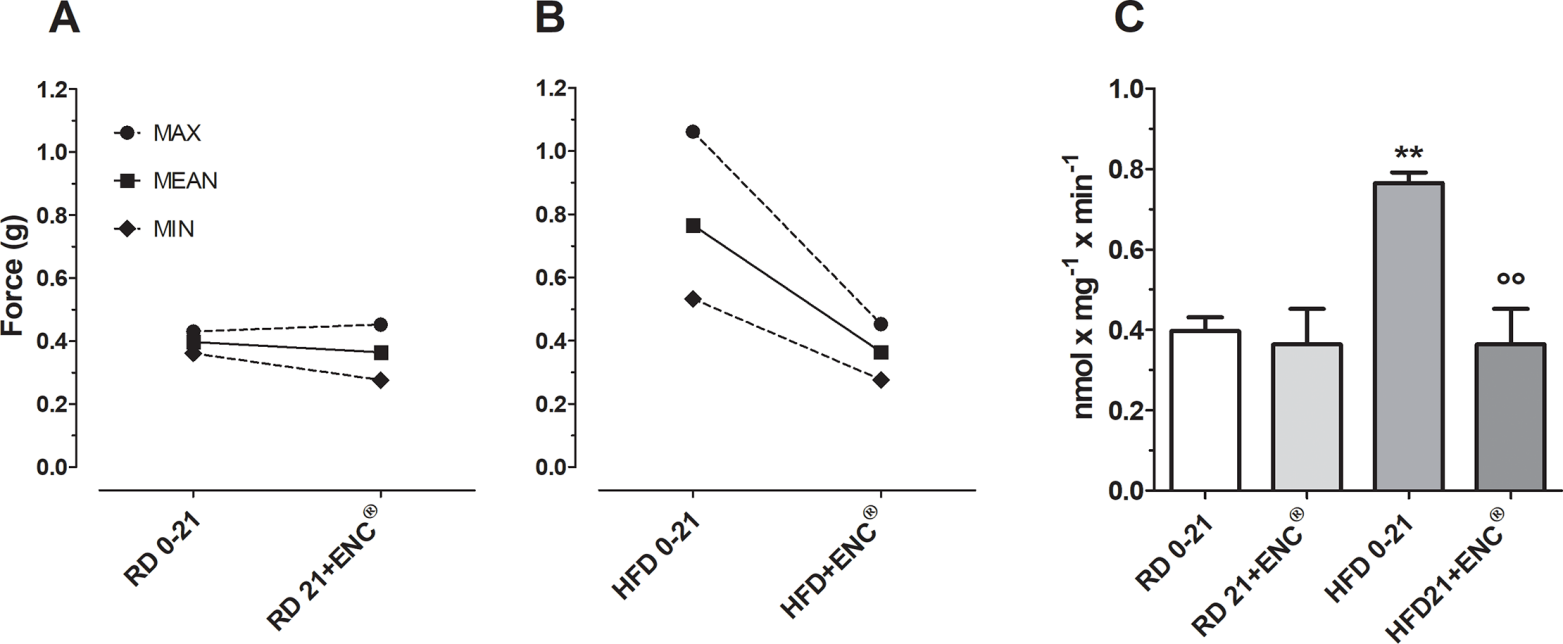
**Basal gastric spontaneous contractility in RD- (panel A) and HFD-rats (panel B) before and after 21 days supplementation with ENC**^**®**^ **(20 mg/kg/day). Panel C: columns comparing the specific values for RD- and HFD-rats.** Data are expressed as mean ± SD. **P < 0.01, *vs* RD same time; °°*P* < 0.01 *vs* HFD0-21 (ANOVA followed by Bonferroni post test).

The gastric spontaneous contractility in HFD-rats was characterized by a higher basal tone than in RD-rats (*P* < 0.01). After ENC^®^ the basal tone was unchanged in RD-rats while in HFD-rats the contraction force was reduced to values within the range of RD-rats, suggesting that 21 days ENC^®^ treatment restored the gastric tonic state to values within the normal range (Fig 10).

#### Induced contractility

Ileum- and distal colon-induced contractility by Carbachol (CCh) and KCl (80 mM) was studied. In RD-fed rats, CCh caused a rapid phasic response, while KCl induced a tonic contraction of rat ileum and proximal colon, as previously reported [25]. Atropine antagonized in concentration dependent manner the response to CCh both in ileum and colon (Table 2 and S3 Table).

**Table 2:** Agonist [carbachol (CCh)] and antagonist (atropine) activities expressed as pEC_50_ or p*A*_2,_ respectively, in ileum and proximal colon of RD- and HFD-rats supplemented with or without ENC^®^ for 21 days.

		RD	RD + ENC^®^^a^	HFD	HFD+ ENC^®^^a^
	Day	0–21	21	0–21	21
**Ileum**	CCh ^**b**^	6.27 ± 0.03	6.34 ± 0.05	5.89 ± 0.02**	5.90 ± 0.04*
	Atropine ^**c**^	8.95 ± 0.03	8.90 ± 0.05	8.68 ± 0.04**	8.91 ± 0.02
**Proximal Colon**	CCh ^**b**^	5.92 ± 0.02	5.80 ± 0.02^##^	5.83 ± 0.03*	5.43 ± 0.02**°°
	Atropine^**c**^	8.77 ± 0.03	8.57 ± 0.04^##^	7.73 ± 0.07***	8.73 ± 0.02**°°

a) RD-and HFD-rats have been given 21 days ENC^®^ (20 mg/kg/day b.w).

b) Data are expressed as pEC_50_. pEC_50_ = –log EC_50_. EC_50_ values are the means ± S.E.M. of at least four independent experiments and were calculated by a non-linear regression curve-fitting computer program [28] [50]

c) Data are expressed as p*A*_2_. p*A*_2_ values ± S.E.M. were calculated from Schild plots [26], constrained to slope –1.0 [27] (p*A*_2_ is the positive value of the intercept of the line derived by plotting log (DR– 1) *vs* log [antagonist]. The log (DR– 1) was calculated from three different antagonist concentrations, and each concentration was tested from four to six times. Dose-ratio (DR) values represent the ratio of the potency of the agonist carbachol (EC_50_) in the presence of the antagonist and in its absence. Parallelism of concentration–response curves was checked by linear regression, and slopes were tested for significance (*P* < 0.05).

*P <0.05

**P < 0.01

***P <0.001 *vs* RD, same time.

°°*P* < 0.01, *vs* HFD0-21.

## *P*< 0.01 vs RD0-21 (ANOVA followed by Bonferroni post test).

**Fig 11:**
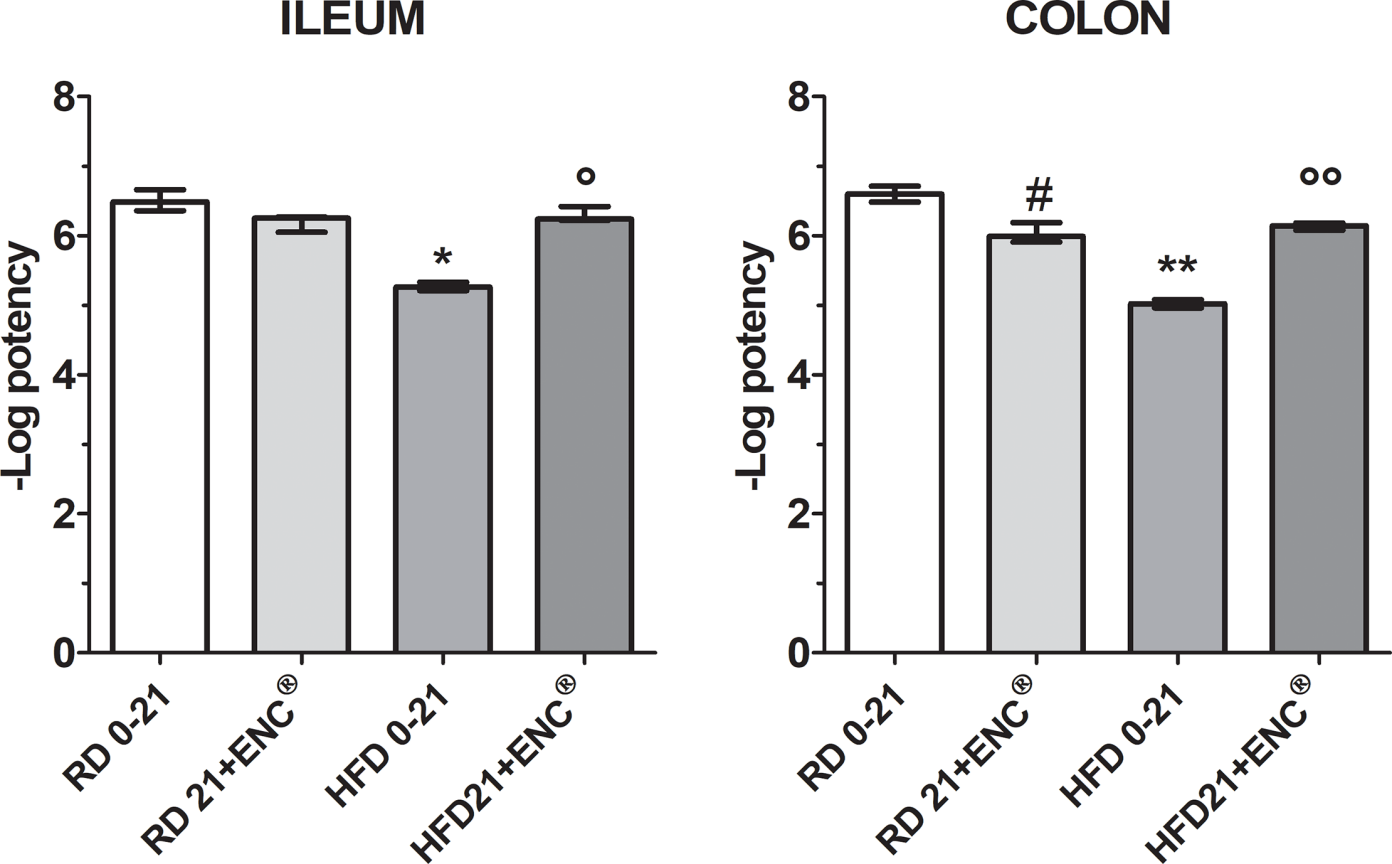
Potency of Nifedipine (pEC_50_) on ileum and distal colon in RD-and HFD-rats with or without ENC^®^ (20 mg/kg/day) for 21 days. The spasmolytic effect induced by Nifedipine was used as reference. Data are expressed as mean ± S.E.M.. *P<0.05, P<0.01 *vs* RD0-21; °P<0.05, °°P<0.01 *vs* HFD 0–21 (ANOVA followed by Bonferroni post test).

In ileum, HFD significantly reduced CCh and atropine pA_2_ (*P* < 0.01) with respect to RD-rats; ENC^®^ decreased CCh pD_2_ difference (*P*<0.05). In ileum ENC^®^ did not change CCh pD_2_ after days 21 treatment in HFD-rats, while atropine pA_2_ values were not different from RD-rats. In RD-rats proximal colon, ENC^®^ slightly reduced CCh potency, less than in the ileum, and Atropine potency. In HFD-rats colon, both CCh and Atropine were less potent than in the same organs of RD-rats. In RD-rat ileum and proximal colon, Nifedipine inhibited tonic contraction induced by 80 mM KCl (Fig 11) (for detailed data, see S3 Table).

After ENC^®^ administration, no significant changes in Nifedipine intrinsic activity and potency were observed in RD-fed rats ileum. In HFD-fed rats ileum, Nifedipine showed the same intrinsic activity, but at concentrations which differ by one order of magnitude (Fig 11) (For detailed data, see S3 Table). Potency on the HFD-fed rats ileum was about 16.39 times lower than on RD-fed rats. After 21 days ENC^®^ treatment, Nifedipine potency was not quite different from that observed in RD-fed rats ileum. In RD-fed rats colon, Nifedipine potency decrease was related to the days of treatment and became lower by 4.04 at the end of the experiment (21 days of treatment). In HFD-fed rats colon, Nifedipine presented intrinsic activity and potency significantly different from RD-fed rats colon. Potency was reduced by 37.72 times. 21 days ENC^®^ treatment of HFD-fed rats increased Nifedipine potency on the colon, which attained values very close to those observed in RD-fed rats colon (Fig 11) (for detailed data, see S3 Table).

## Discussion

Polyphenols have been shown to exert several beneficial effects in different obesity experimental models [16, 20, 29–31], by scavenging reactive oxygen and nitrogen species and modulating genes associated with metabolism, drug metabolizing enzymes, detoxification proteins, protecting from chronic diseases [32].

A down-regulation of liver oxidative defenses has been reported in obesity experimental models [33]. Consistently, in the present work, ENC^®^ restored the antioxidant enzymatic ability in HFD-rats and suppressed pro-inflammatory / anti-inflammatory cytokines imbalance in HFD rat liver. A crucial point is the decrease of TNF-α, a key factor in the development of Non Alcoholic Fatty Liver Disease (NAFLD) and Non Alcoholic Steato Hepatitis (NASH) [34], of IL-6, hepatoprotector in liver steatosis by reducing oxidative stress [35], of IL-10 [36] and of IL-17A, implicated in both regulation of obesity and NAFLD [37].

NAFLD is characterized by a general dysregulation of drug metabolism enzymes. Several studies reported that GSTM1, GSTM2, GSTM4 and GSTM5 mRNA levels are suppressed in patients with steatosis and NASH [38]. Moreover, GST activity decreases with disease progression with a reduced pool of glutathione, highlighting the reduced ability to counteract OS in these patients [39].

Finally, GSTM1-null genotype has been associated to NAFLD [40] and UDPGT levels were decreased in HFD-fed rats [41]. In HFD-rats ENC^®^ increased enzymatic activity of GST and UDPGT and it can reduce the dysregulation of drug metabolism enzymes. Moreover, these finding could be clinically relevant for obese patients who have additional comorbidities and are receiving multiple medications.

These outcomes are extremely important in a therapeutic schedule of obese patients at possible risk of NAFLD and NASH. ENC^®^ slightly reduced liver steatosis induced by HFD. Since flavonoids induce a cytoprotective effect through Nrf2-dependent gene expression [42] we think that up-regulation of antioxidant and detoxifying enzymes is obtained of Nrf2-ARE signalling pathway. Intracellular accumulation of xeno- and endobiotics is regulated at several levels by drug metabolizing enzymes.

Many tannins possess anti-obesity properties [43]. Pomegranate leaf extract containing abundant tannins, for example, can hamper the development of obesity and hyperlipidemia in high-fat diet induced obese mice by inhibiting the pancreatic lipase activity and suppressing energy intake [44]. This effect was much less evident when the animals were treated with its main isolated compounds (ellagic acid and tannic acid), suggesting a possible synergic effects among the component of the extract. Furthermore, growing number of studies demonstrate the potential role of ellagic acid in the regulation of lipid metabolism and in attenuating obesity and metabolic syndrome in rodents, mainly because of its antioxidant and anti-inflammatory properties (for a review see [45]). Thus it is possible that all the properties reported for ellagic acid are shared also by ENC^®^ or its gut metabolites. Is is important to outline, however, that the amount of ellagic- and gallic-acid contained in ENC^®^ is ~ 1–2% of the injected dose, suggesting that other components may contribute to the observed effects. It is possible that other polyphenols found in ENC^®^ might act synergistically by increasing, for example, the potency of ellagic and gallic acids at its specific molecular target(s) or by increasing the bioavailability of the main active components. In this sense, the literature already reports the existence of synergistic effects among several phytochemicals [46].

Obesity has been correlated with an induction of some cytochrome P450 isoforms (CYPs) [5, 47, 48]. The NADPH-dependent overproduction of reactive oxygen species (ROS) is linked to CYP induction [49].

In HFD-rats an up-regulation of mainly CYP2E1, CYP3A1 and CYP2B1/2 CYPs has been found. CYP2E1 values were reduced by four times by ENC^®^. CYP2E1 up-regulation has been associated to obesity, fatty liver, and NASH in both humans and rodents and correlates with NAFLD severity [50] and progression of liver steatosis to NASH: CYP2E1 up-regulation leads to enhanced ROS production, with an increase of lipid peroxidation and inflammation [51].

HFD led to a general impairment of cellular redox homeostasis responsible for the observed alterations of phase I and II antioxidant enzymes. We focused on the peroxidation products in intestinal tissues from all experimental groups. In RD-rats ileum, ENC^®^ increased MDA levels, likely due to the high amount of ellagitannins, that are slowly hydrolysed in the digestive tract, to release ellagic acid. In the colon OS was increased, as observed in the mouse distal colon of HFD mice [52].

The compound inhibited HFD-induced oxidative stress increase, in agreement with published data showing that polyphenols rich extracts provide benefits in gut inflammation, for their antioxidant activity, due to a prebiotic [53] action that may help reduce OS in obese patients.

Since obesity seems associated to increased gastric emptying, we evaluated the basal gastric tone of HFD- and RD-rats. ENC^®^ restored the gastric tone in HFD-fed rats. HFD alters the composition of cells membranes, resulting in an impaired response to endogenous agonists such as Ach and histamine; also intracellular calcium release may be disturbed. In a previous work, we demonstrated that ENC^®^ exerts a non-competitive and concentration-dependent antagonist effect towards muscarinic and histaminergic receptors and inhibits BaCl_2_-induced contractions: these effects may be, at least in part, responsible for ENC^®^ beneficial effects towards gastric smooth muscle contractility alterations related to obesity [21]. To evaluate the intestinal cell membrane functionality, we used CCh and atropine as a muscarinic receptor agonist and antagonist respectively, and nifedipine as calcium channel blocker. ENC^®^ spasmolytic effect depends on a combination of effects towards different receptors and channels. KCl induced tonic contraction on rat ileum and colon while CCh caused a rapid phasic contraction. Nifedipine completely inhibited K^+^ induced contraction. In RD-rats, ENC^®^ (21 days) reduced nifedipine potency by 4.04 times in the colon, with a minimal decrease in the ileum (by 1.69 times). Hypercholesterolemic diet has been reported to decrease calcium channel pump activity in pigs coronary macrocirculation [54] and cholesterol enriched cyclodextrin inhibited calcium currents in guinea pig gallbladder smooth muscle [55]. In HFD-rats, nifedipine potency was 16.39 and 37.72 times less potent than in RD-rats ileum and colon, respectively. Importantly, ENC^®^ decreased the activity of L-type calcium channels in RD-rats mainly in colon, but increased their function mostly in the HFD-rats colon. ENC^®^ decreases nifedipine potency in ileum and more strongly in colon. Therefore, in HFD-rats calcium channels function progressively recovers after ENC^®^ administration.

The activity and responsiveness of calcium channels in HFD-fed rats may be worthwhile considering. The induced increase in calcium channels activity may influence the basal tone, affect contractility and alter the response to receptor agonists and antagonists. ENC^®^ showed a not competitive and reversible muscarinic effect on isolated ileal and colonic muscular strips and ENC^®^ restored receptors function in HFD-rats. Also in the case of membrane coupled receptors, the observed increase of the activity showed the restoration of membrane function, that supported the ENC^®^ effects on basal gastric contractility.

## Conclusions

In conclusion, our study shows that ENC^®^ may be a promising nutraceutical tool, decreasing spontaneous food assumption, up-regulating hepatic antioxidant enzymes causing the impairment in liver oxidative defenses, restoring liver phase I and II enzymes activity, reducing intestinal oxidative stress, and restoring the intestinal motor function. These ameliorative effects on metabolic situation in obesity might be related to the presence of ellagic and gallic acid. As recently reported on hypertriglyceridemia induced by High-Fructose Diet in rats, ENC^®^ contains such gallic acid equivalents as to decrease hypercholesterolemia induced by HFD [56]. All together these results show that ENC^®^ has the potential to be used in clinical medicine as a dietary supplement in the treatment of obesity complications.

## Supporting information

S1 File Chemicals. (DOCX)

S2 File High fat diet. (DOCX)

S3 File Humane end points.docx. (DOCX)

S4 File Methods. (DOCX)

S1 Table Liver histology of RD- or HFD-rats supplemented with or without ENC^®^ for 21 days. (DOCX)

S2 Table Effects of 21 days ENC^®^ supplementation (20 mg/kg/day) for 21 days on liver phase I (A), phase II (B) and antioxidant (C) enzymes activities in RD and HFD rats.
(DOCX)

S3 Table Agonist [carbachol (CCh)] and antagonist (atropine) activities expressed as pEC_50_ or pA_2,_ respectively, (A) and Calcium channels antagonist Nifedipine activity (B) in the isolated rat ileum and distal colon of RD- and HFD-rats supplemented with or without ENC^®^ (20 mg/kg/day) for 21 days.
(DOCX)
