# Supplementary material for: Liver and intestinal protective effects of Castanea sativa Mill. bark extract in high-fat diet rats
Source: PLoS One. 2018 Aug 6;13(8):e0201540. doi: 10.1371/journal.pone.0201540 (PMC6078294; doi:10.1371/journal.pone.0201540)
Supplement: S1 File — (DOCX) [file pone.0201540.s001.docx]

**S1 Chemicals**

Nicotinamide adenine dinucleotide phosphate in oxidized and reduced form (NADP^+^ and NADPH), sodium dithionite, 7-ethoxyresorufin, *p-*nitrophenol, aminopyrine, dichlorophenolindophenol (DCPIP), phenylmethylsulfonyl fluoride, bovin serum albumin, ethoxycoumarin, pentoxyresorufin, methoxyresorufin, resorufin, L-glutathione reduced, 1-chloro-2,4-dinitrobenzene, 1-naphtol, Triton X-100, epinephrine, Benzene-1,2-dithiol, Folin-Ciocalteu reagent, (±)-6-hydroxy-2,5,7,8-tetramethylchromane- 2-carboxylic acid (Trolox), cytocrome C, isocytrate, isocytrate DH, Umbelliferone, uridine-5-diphoshoglucuronic acid were purchased from Sigma-Aldrich (St. Luis, MO, USA); glusose 6-phosphate and glucose 6-phosphate dehydrogenase from Roche Diagnostic (Indianapolis, IN, USA).

All others chemicals were highest purity commercially available.
