# Supplementary material for: Liver and intestinal protective effects of Castanea sativa Mill. bark extract in high-fat diet rats
Source: PLoS One. 2018 Aug 6;13(8):e0201540. doi: 10.1371/journal.pone.0201540 (PMC6078294; doi:10.1371/journal.pone.0201540)
Supplement: S2 File — (DOCX) [file pone.0201540.s002.docx]

**S2 High Fat Diet**

Animals were fed a fat rich diet, providing 60% energy from lipids (Mucedola, s.r.l. Via Galileo Galilei, 6, Settimo Milanese MI, Italy). In Table S1 the main components are reported. For more detailed information, see www.mucedola.it/.

**Composition of high fat diet**

| **INGREDIENTS** | **ADDITIVES per kg** | | | |
| --- | --- | --- | --- | --- |
|  | ***Nutritional additives*** | | ***Preservative*** | |
| Casein powder | Vitamin A | I.U. 8400 | Potassium citrate | |
| Sucrose | Vitamin D3 | I.U. 2000 |  | |
| Lard | Fe | mg 55 | ***Colorants*** | |
| Maltodextrin | Mn | mg 14.5 | Blue patent V (E131) | mg 50 |
| Sucrose | Zn | mg 46 |  | |
| Palm oil | Cu | mg 8,2 |  | |
| Soybean oil | I | mg 0,29 | **Analysis constituents %** | |
| Calcium carbonate | Se | mg 0,20 | Crude Protein | 23.00 |
| Sodium chloride | Mo | mg 0.21 | Crude oils and Fats | 34.00 |
| Mineral dicalcium phosphate |  | | Crude Fibres | 5.00 |
| Magnesium oxide |  | | Crude ash | 5.50 |
