# Supplementary material for: Liver and intestinal protective effects of Castanea sativa Mill. bark extract in high-fat diet rats
Source: PLoS One. 2018 Aug 6;13(8):e0201540. doi: 10.1371/journal.pone.0201540 (PMC6078294; doi:10.1371/journal.pone.0201540)
Supplement: S3 File — (DOCX) [file pone.0201540.s003.docx]

**S3 File Humane End Points**

For the studies in which death of the animals were a planned experimental endpoint, the following information was reported about:

Humane endpoints used to determine when animals should be euthanized.

The duration of the experiment.

The numbers of animals used, euthanized

Animal health and behavior monitoring.

Efforts to minimize suffering and distress, use of special housing conditions were used.

All data according to: <https://www.humane-endpoints.info/en>
