# Supplementary material for: Liver and intestinal protective effects of Castanea sativa Mill. bark extract in high-fat diet rats
Source: PLoS One. 2018 Aug 6;13(8):e0201540. doi: 10.1371/journal.pone.0201540 (PMC6078294; doi:10.1371/journal.pone.0201540)
Supplement: S4 File — (DOCX) [file pone.0201540.s004.docx]

**S4 Methods**

**Determination of Serum lipids and transaminase**

Triglyceride, HDL-cholesterol and total cholesterol concentrations were measured using Dimension RxL Max system Kit (Siemens Healthcare Diagnostics, Newark, DE, USA), following manufacturer’s instructions. Serum transaminases were evaluated by Sigma-Aldrich colorimetric assay. For each sample a volume of 200 µl was used.

**Plasma Inflammatory Response**

One commercially available kit was used for the simultaneous quantification of IL-1α, L-1β, IL-2, IL-4, IL-5, IL-6, IL-7, IL-10, IL-12p70, IL-13, IL-17A, IL-18, and TNF-α (Bio-Rad, Bio-Plex Pro™ Rat Cytokine 24-plex Assay #171K1001M). Plasma samples were collected as 2 mL aliquots from each rat at the beginning and at the end of the study (not at each time point) Standard solutions were prepared for the calibration curve and quality controls according to the manufacturer’s protocol. Briefly, seven standards obtained by serial dilutions, the quality controls, 100 µl of plasma and the magnetic beads were loaded into a 96-well plate and incubated overnight to allow the antibody and analyte of interest to form a bond. The plate was washed three times and then 50 μl of a cocktail of the specific biotinylated antibodies was added to each well and incubated for 1 h. Next, the plate was washed again three times to remove unbound biotinylated antibodies, then streptavidin-phycoerythrin conjugate (streptavidin-PE) was added to each well. Another wash was then performed to remove unbound streptavidin-PE and the beads were resuspended in 100 µl of Sheath Fluid solution. Finally, the plates were read through the MAGPIX Luminex® system and data were analyzed using the Analyst 5.1 PLUS software (Milliplex^®^).

**Liver histology**

Histopathology. Ten mg of liver tissues were taken from freshly sacrificed animals. Slices were fixed in 4% formaldehyde for histopathological analysis. Paraffin sections (1 µm thick) were stained with hematoxylin and eosin. Liver histology was evaluated in blinded fashion by two expert pathologists and necroinflammation and architectural changes, fibrosis and cirrhosis staging were scored according to [1],

**Liver Histology Score According To Ishak [1]**

| **Necroinflammatory Scores** | | | | | | | |
| --- | --- | --- | --- | --- | --- | --- | --- |
| **Periportal or Periseptal Interface Hepatitis (piecemeal necrosis)** | **Score** | **Confluent**  **Necrosis** | **Score** | **Focal (spotty) Lytic Necrosis, Apoptosis and Focal Inflammation** | **Score** | **Portal**  **Inflammation** | **Score** |
| Absent | 0 | Absent | 0 | Absent | 0 | None | 0 |
| Mild (focal, few portal areas) | 1 | Focal confluent necrosis | 1 | One focus or less per 10x objective | 1 | Mild, some or all portal areas | 1 |
| Mild/moderate (focal, most portal areas) | 2 | Zone 3 necrosis in some areas | 2 | Two to four foci per 10x objective | 2 | Moderate, some or all portal areas | 2 |
| Moderate (continuous around <50% of tracts or septa) | 3 | Zone 3 necrosis in most areas | 3 | Five to ten foci per 10x objective | 3 | Moderate/marked, all portal areas | 3 |
| Severe (continuous around >50% of tracts or septa) | 4 | Zone 3 necrosis + occasional portal-central (P-C) bridging | 4 | More than ten foci per 10x objective | 4 | Marked, all portal areas | 4 |
|  |  | Zone 3 necrosis + multiple P-C bridging | 5 |  | | | |
|  |  | Panacinar or multiacinar necrosis | 6 |  |  |  |  |

*Does not include diffuse sinusoidal infiltration by inflammatory cells

**Staging: Liver Architectural Changes, Fibrosis And Cirrhosis**

| **Modified Staging: architectural changes, fibrosis and cirrhosis** | |
| --- | --- |
| **Change** | **Score** |
| No fibrosis | 0 |
| Fibrous expansion of some portal areas, with or without short fibrous septa | 1 |
| Fibrous expansion of most portal areas, with or without short fibrous septa | 2 |
| Fibrous expansion of most portal areas, with occasional portal to portal (P-P) bridging | 3 |
| Fibrous expansion of portal areas with marked bridging [portal to portal (P-P) as well as portal to central (P-C)] | 4 |
| Market bridging (P-P and/or P-C) with occasional modules (incomplete cirrhosis) | 5 |
| Cirrhosis, probable or definite | 6 |

**Liver collection and preparation of subcellular fractions**

### The liver collection and preparation of subcellular fractions has been performed as previously described [2]. Suitable concentrations and others details were previously reported [3]. The protein concentration of the microsomal and cytosolic fractions was determined according to the method described by Lowry et al. [4] as revised by Bailey [5]. The enzymatic assays described below were performed on the liver microsomal fraction.

**Phase I enzymes activities**

NADPH-(CYP)-c-reductase (CYP-red): The analytical method is based on the determination of the reduction rate of cytochrome c at 550 nm (ε = 19.1 mM^-1^ cm^-1^), according to previously defined procedures [6]. Incubation mixture contained 1.6 ml of 0.05 M Tris-HCl buffer (pH = 7.7) with 0.1 mM EDTA, 0.5 mg cytochrome c, 0.2 ml of microsomes. Reaction begins with addiction of 0.2 ml NADPH. Specific reaction was read at 550 nm against buffer plus cytochrome c.

*Aminopyrine N-demethylase (APND)-CYP3A1/2*. Activity was determined by quantification of CH2O release, according to [7]. The total incubation volume was 3 ml, composed of 0.5 ml of water solution of 50 mM aminopyrine and 25 mM MgCl_2_, 1.48 ml of 0.60 mM NADP^+^, 3.33 mM G6P in 50 mM Tris-HCl buffer (pH 7.4), 0.02 ml G6PDH (0.93 U/ml) and 0.125 ml of sample. After 5 minutes of incubation at 37 °C, the yellow color developed by the reaction of the released of CH_2_O with the Nash reagent, was read at 412 nm, and the molar absorptivity of 8,000 at 412 nm used for calculation [3, 8].

p-Nitrophenol hydroxylase (p-NPH)-CYP2E1*:* Activity was determined in a final volume of 2 ml: 2 mM p-nitrophenol in 50 mM Tris-HCl buffer (pH 7.4), 5 mM MgCl_2_, and a NADPH-generating system consisting of 0.4 mM NADP^+^, 30 mM isocytrate, 0.2 U of isocytrate dehydrogenase and 1.5 mg of proteins. After 10 minutes of incubation at 37 °C, the reaction was terminated by addition of 0.5 ml of 0.6 N perchloric acid. Precipitated proteins were removed by centrifugation and 1 ml of the resultant supernatant was mixed with 1 ml of 10 N NaOH. Absorbance at 546 nm was immediately recorded and 4-nitrocathecol determined (ε = 10.28 mM^-1^ cm^-1^) [9, 10].

Pentoxyresorufin O-dealkylase (PROD)-CYP2B1/2, ethoxyresorufin O-deethylase, (EROD)-CYP1A1 and methoxyresorufin O-demethylase (MROD)-CYP1A2: Reaction mixture (PROD) consisted of 0.025 mM MgCl_2_, 200 mM pentoxyresorufin, 0.32 mg of proteins and 130 mM NADPH in 2.0 ml 0.05 M Tris-HCl buffer (pH 7.4). Resorufin formation at 37 °C was calculated by comparing the rate of increase in relative fluorescence to the fluorescence of known amounts of resorufin (excitation 563 nm, emission 586 nm) [11]. EROD and MROD activities were measured exactly in the same manner as described for the pentoxyresorufin assay, except that substrate concentration was 1.7 mM for ethoxyresorufin and 5 mM for methoxyresorufin [12, 13].

Ethoxycoumarin O-deethylase (ECOD)-CYP1A1/2, CYP2A, CYP2B, CYP2E1: ECOD was determined by the quantification of umbelliferone formation, according to Aitio [14]. Incubation mixture consisted in 2.6 ml, composed of 1 mM ethoxycoumarin, 5mM MgCl_2_, NADPH-generating system (see aminopyrine assay) and 0.25 ml of sample. After 5 minute of incubation at 37 °C, reaction was stopped by the addiction of 0.85 ml of trichloroacetic acid 0.31 M. The pH of the mixture was brought to about 10 by adding 0.65 ml of 1.6 M NaOH-glycine buffer (pH = 10.3); the amount of umbelliferone was measured fluorimetrically (excitation 390 nm; emission 440 nm) [15].

**Phase II enzyme activities**

*Glutathione S-transferase (GST) activity*: The incubation mixture for measuring overall GST activity contained 1 mM glutathione, 1 mM 1-chloro-2,4-dinitrobenzene (CDNB) in methanol, 0.025 ml of sample in a final volume of 2.5 ml 0.1 M phosphate Na^+^/K^+^ buffer (pH 6.5). The product of the reaction of the thiol group of glutathione with the electrophilic group of CDNB was read at 340 nm (ε= 9.6 mM^-1^ cm^-1^)] [16].

*UDP-glucuronosyl transferase (UDPGT) activity*: The overall UDPGT activity was determined kinetically using 1-naphtol as substrate (final concentration, 50 mM) by the continuous fluorimetric (excitation 390 nm; emission 440 nm) monitoring of 1-naphtholglucuronide production in the presence of 1 mM uridine-5’-diphosphoglucuronic acid [17]. Experiments were performed in the presence or absence of Triton X-100 (0.2%) as a detergent, in order to improve the assay sensitivity [18].

**Antioxidant enzyme activities**

Catalase (CAT) activity: The reaction was started in a quartz cuvette, containing 50 mM potassium phosphate buffer and cytosol sample, by adding 30 mM H_2_O_2_. The decomposition of the substrate was measured at 240 nm and catalase activity was expressed as mol H_2_O_2_ consumed per minute per mg protein using a molar extinction coefficient of 43.6 mM^-1^cm^-1^ [19, 20].

NAD(P)H:quinone reductase (NQO1) activity: NQO1 activity was assayed spectrophotometrically at 600 nm by monitoring the reduction of the blue redox dye of DCPIP (ε = 9.6 mM^-1^ cm^-1^), and expressed as mol DCPIP reduced per minute per mg protein [19, 21].

Oxidised glutathione reductase activity (GSSG-red): GSSG-red activity was measured by adding 1.5mM NADPH to an assay cuvette containing 50 mM potassium phosphate buffer, 1 mM EDTA, cytosol sample and 20mM GSSG. The generation of NADP^+^ from NADPH, during the reduction of GSSG, was recorded at 340 nm for 5 min at 37 °C. GSSG-red activity was calculated using the extinction coefficient of 6.22 per mM^-1^ cm^-1^, and expressed as mol NADPH consumed per min per mg protein [19].

Superoxide dismutase activity (SOD): SOD activity was determined according to Misra HP and Fridovich I assay [22]. Briefly, the activity was assayed spectophotometrically at 320 nm by monitoring the generation of adenochrom, one of main products of epinephrine autoxidation at pH 10.2. The dejection of autooxidation was used to calculate SOD activity, by using the extinction coefficient of 4.02 per mM^-1^cm^-1^ and expressed as mol of epinephrine oxidized per min per mg protein, derived by subtracting each test curves from the epinephrine autoxidation standard [19].

**Determination of OXIDATIVE stress in the ileal and colonic tissues**

Thiobarbituric acid (TBA) assay of malondialdehyde (MDA): colon and ileum were homogenized in distilled water by means of an ultra-turrax homogenizer. Quantification of TBA reactive substances (TBARS) was carried out as described by Buege and Aust [23].

**Functional in vitro studies**

Gastric Fundus spontaneous contractility: The stomach was removed, opened along the mesentery of the greater curvature and rinsed with Krebs-bicarbonate buffered solution of the following composition (mM): NaCl, 120; KCl, 4.6; CaCl_2_, 2.5; MgCl_2_, 1.2; NaH_2_PO_4_•2 H_2_O, 1.2; NaHCO_3_ 22; glucose 11.5; maintained at 37 °C and gassed with 95% O_2_ and 5% CO_2_. The mucosa was removed from the mid-fundus. Fundus muscle strips were cut parallel to the circular muscle layer, as seen by the muscle fibre direction of the upper most muscle layer. These strips were then tied at each end with sutures and hung in 15 ml organ baths containing Krebs-bicarbonate buffered solution. The spontaneous contractility was continuously recorded with the LabChart 7 PRO Software (AD Instruments, Bella Vista, New South Wales, Australia). After the equilibration period (about 30 to 45 min according to each tissue), the following parameters were evaluated considering a 5 min stationary period. All the calculations were performed in a post-processing phase by using Lab Chart Software. In order to avoid errors due to the presence of artifacts, the period of analysis was chosen by a skilled operator.

Ileum and proximal colon induced contractility**:** The terminal portion of ileum (immediately proximal to the ileo-caecal junction) was cleaned, and 1 cm segments were used. One cm segment of the proximal colon was transected and the mesenteric tissue was removed. The tissues were rinsed and mounted in 15-ml organ bath containing Tyrode solution of the following composition (mM): NaCl, 118; KCl, 4.47; CaCl_2_•2H_2_O, 2.54; MgSO_4_, 1.2; KH_2_PO_4_•2H_2_O, 1.19; NaHCO_3_ 25; glucose 11. The physiological salt solution (PSS) was buffered at pH 7.4 by saturation with 95% O_2_ – 5% CO_2_ gas, and the temperature was maintained at 35 °C. Each segment was mounted longitudinally under a resting tension of or 1g. Tissues were allowed to equilibrate for 60 min during which time the bathing solution was changed every 10 min. After an equilibration period (60 - 90 min), the tissues were used to test spontaneously and carbachol induced contraction.

Cholinergic activit*y*: Appropriate tension was applied to each muscle strip, then equilibrated for 1 h. Concentration-response curves were constructed by cumulative addition of the agonist carbachol (CCh). The concentration of agonist in the organ bath was added only after the response to the previous addition had attained a maximal level and remained steady. Contractions were recorded by means of displacement transducer (FT. 03, Grass Instruments, Quincy, MA) using Power Lab software (ADInstruments Pty Ltd, Castle Hill, Australia). Concentration–response curves to agonist were obtained at 30 min intervals, the first one being discarded and the second one used as control. Following incubation with the antagonist (Atropine) for 30 min, a new concentration-response curve to agonist was obtained. Tension changes were recorded isotonically. In all cases, parallel experiments in which tissues did not receive any antagonist were run in order to check any variation in sensitivity. It was always verified that the EC_50_ values for the agonist in tissues receiving only the solvent were not significantly different (*P* > 0.05) from the control values. In all other cases experiments were discarded. Functional activity of CCh and antagonism of atropine *vs* CCh-induced contraction, was determined in gut segments taken from different groups of rats.

Calcium channels antagonistic activity*:* The isolated ileum and proximal colon segments were prepared as previously described and placed under a resting force of 1 g and washed every 20 min with fresh Tyrode solution (see above) for 1 h. After the equilibration period, tissues were contracted by washing in PSS containing 80 mM KCl (equimolar substitution of K^+^ for Na^+^). When the contraction reached a plateau (about 45 min) various concentrations of nifedipine were added cumulatively to the bath allowing for any relaxation to obtain an equilibrated level of force.

**References**

1. Ishak KG, Zimmerman HJ, Ray MB. Alcoholic liver disease: pathologic, pathogenetic and clinical aspects. Alcohol Clin Exp Res. 1991;15(1):45-66. PubMed PMID: 2059245.

2. Canistro D, Vivarelli F, Cirillo S, Costa G, Andreotti C, Paolini M. Comparison between in toto peach (Prunus persica L. Batsch) supplementation and its polyphenolic extract on rat liver xenobiotic metabolizing enzymes. Food Chem Toxicol. 2016;97:385-94. doi: 10.1016/j.fct.2016.10.006. PubMed PMID: 27742397.

3. Melega S, Canistro D, De Nicola GR, Lazzeri L, Sapone A, Paolini M. Protective effect of Tuscan black cabbage sprout extract against serum lipid increase and perturbations of liver antioxidant and detoxifying enzymes in rats fed a high-fat diet. Br J Nutr. 2013;110(6):988-97. doi: 10.1017/S0007114513000068. PubMed PMID: 23433361.

4. Lowry OH, Rosebrough NJ, Farr AL, Randall RJ. Protein measurement with the Folin phenol reagent. J Biol Chem. 1951;193(1):265-75. PubMed PMID: 14907713.

5. Bailey JL. Techniques in protein chemistry. 2d rev. and expanded ed. Amsterdam, New York,: Elsevier Pub. Co.; 1967. xiv, 406 p. p.

6. Canistro D, Melega S, Ranieri D, Sapone A, Gustavino B, Monfrinotti M, et al. Modulation of cytochrome P450 and induction of DNA damage in Cyprinus carpio exposed in situ to surface water treated with chlorine or alternative disinfectants in different seasons. Mutat Res. 2012;729(1-2):81-9. doi: 10.1016/j.mrfmmm.2011.09.008. PubMed PMID: 22001235.

7. La Du BN, Mandel HG, Way EL. Fundamentals of drug metabolism and drug disposition. Baltimore,: Williams & Wilkins Co.; 1971. xvii, 615 p. p.

8. Nash T. The colorimetric estimation of formaldehyde by means of the Hantzsch reaction. Biochem J. 1953;55(3):416-21. PubMed PMID: 13105648; PubMed Central PMCID: PMCPMC1269292.

9. Canistro D, Barillari J, Melega S, Sapone A, Iori R, Speroni E, et al. Black cabbage seed extract affects rat Cyp-mediated biotransformation: organ and sex related differences. Food Chem Toxicol. 2012;50(8):2612-21. doi: 10.1016/j.fct.2012.05.030. PubMed PMID: 22634264.

10. Reinke LA, Moyer MJ. p-Nitrophenol hydroxylation. A microsomal oxidation which is highly inducible by ethanol. Drug Metab Dispos. 1985;13(5):548-52. PubMed PMID: 2865101.

11. Lubet RA, Mayer RT, Cameron JW, Nims RW, Burke MD, Wolff T, et al. Dealkylation of pentoxyresorufin: a rapid and sensitive assay for measuring induction of cytochrome(s) P-450 by phenobarbital and other xenobiotics in the rat. Arch Biochem Biophys. 1985;238(1):43-8. PubMed PMID: 3985627.

12. Burke MD, Thompson S, Elcombe CR, Halpert J, Haaparanta T, Mayer RT. Ethoxy-, pentoxy- and benzyloxyphenoxazones and homologues: a series of substrates to distinguish between different induced cytochromes P-450. Biochem Pharmacol. 1985;34(18):3337-45. PubMed PMID: 3929792.

13. Sapone A, Canistro D, Vivarelli F, Paolini M. Perturbation of xenobiotic metabolism in Dreissena polymorpha model exposed in situ to surface water (Lake Trasimene) purified with various disinfectants. Chemosphere. 2016;144:548-54. doi: 10.1016/j.chemosphere.2015.09.022. PubMed PMID: 26397472.

14. Aitio A. A simple and sensitive assay of 7-ethoxycoumarin deethylation. Anal Biochem. 1978;85(2):488-91. PubMed PMID: 565602.

15. Vivarelli F, Canistro D, Sapone A, De Nicola GR, Babot Marquillas C, Iori R, et al. Raphanus sativus cv. Sango Sprout Juice Decreases Diet-Induced Obesity in Sprague Dawley Rats and Ameliorates Related Disorders. PLoS One. 2016;11(3):e0150913. doi: 10.1371/journal.pone.0150913. PubMed PMID: 26987061; PubMed Central PMCID: PMCPMC4795736.

16. Vivarelli F, Canistro D, Franchi P, Sapone A, Vornoli A, Della Croce C, et al. Disruption of redox homeostasis and carcinogen metabolizing enzymes changes by administration of vitamin E to rats. Life Sci. 2016;145:166-73. doi: 10.1016/j.lfs.2015.12.033. PubMed PMID: 26702769.

17. Mackenzie PI, Hanninen O. A sensitive kinetic assay for UDPglucuronosyltransferase using 1-naphthol as substrate. Anal Biochem. 1980;109(2):362-8. PubMed PMID: 6784601.

18. Canistro D, Pozzetti L, Sapone A, Broccoli M, Bonamassa B, Longo V, et al. Perturbation of rat hepatic metabolising enzymes by folic acid supplementation. Mutat Res. 2008;637(1-2):16-22. doi: 10.1016/j.mrfmmm.2007.06.007. PubMed PMID: 17681554.

19. Bonamassa B, Canistro D, Sapone A, Vivarelli F, Vornoli A, Longo V, et al. Harmful effects behind the daily supplementation of a fixed vegetarian blend in the rat model. Food Chem Toxicol. 2016;97:367-74. doi: 10.1016/j.fct.2016.09.033. PubMed PMID: 27697540.

20. Wheeler CR, Salzman JA, Elsayed NM, Omaye ST, Korte DW, Jr. Automated assays for superoxide dismutase, catalase, glutathione peroxidase, and glutathione reductase activity. Anal Biochem. 1990;184(2):193-9. PubMed PMID: 2327564.

21. Ernster L, Danielson L, Ljunggren M. DT diaphorase. I. Purification from the soluble fraction of rat-liver cytoplasm, and properties. Biochim Biophys Acta. 1962;58:171-88. PubMed PMID: 13890666.

22. Misra HP, Fridovich I. The role of superoxide anion in the autoxidation of epinephrine and a simple assay for superoxide dismutase. J Biol Chem. 1972;247(10):3170-5. PubMed PMID: 4623845.

23. Buege JA, Aust SD. Microsomal lipid peroxidation. Methods Enzymol. 1978;52:302-10. PubMed PMID: 672633.

24. Motulsky H, Christopoulos A. Fitting models to biological data using linear and nonlinear regression : a practical guide to curve fitting. Oxford ; New York: Oxford University Press; 2004. 351 p. p.

25. Arunlakshana O, Schild HO. Some quantitative uses of drug antagonists. Br J Pharmacol Chemother. 1959;14(1):48-58. PubMed PMID: 13651579; PubMed Central PMCID: PMCPMC1481829.

26. Tallarida RJ, Murray RB. Manual of pharmacologic calculations with computer programs. 2nd ed. New York: Springer-Verlag; 1987. x, 297 p. p.
