# Supplementary material for: Liver and intestinal protective effects of Castanea sativa Mill. bark extract in high-fat diet rats
Source: PLoS One. 2018 Aug 6;13(8):e0201540. doi: 10.1371/journal.pone.0201540 (PMC6078294; doi:10.1371/journal.pone.0201540)
Supplement: S1 Table — (DOCX) [file pone.0201540.s005.docx]

**Table S1.** **Liver histology of RD- or HFD-rats supplemented with or without ENC^®^ for 21 days**

|  | **RD** | **RD + ENC^®^** | | | **HFD** | **HFD+ ENC^®^** | | |
| --- | --- | --- | --- | --- | --- | --- | --- | --- |
| **Day** | **0-21** | **7** | **14** | **21** | **0-21** | **7** | **14** | **21** |
| **Liver histopathological results** | Normal | Normal | Normal | Normal | Steatosis  < 10% | Steatosis  <5% | Micro-macro vescicular steatosis, 5-10% | Micro-macro vescicular steatosis, <5% in 3 zone |
| **Necroinflammatory Score** | 0 | 0 | 0 | 0 | 0 | 0 | 0 | 0 |

To improve clarity, since values between RD 0 day and RD 21 days did not change significantly, they have been pooled; the same was for HFD 0 day and HFD 21 days.
