# Supplementary material for: Liver and intestinal protective effects of Castanea sativa Mill. bark extract in high-fat diet rats
Source: PLoS One. 2018 Aug 6;13(8):e0201540. doi: 10.1371/journal.pone.0201540 (PMC6078294; doi:10.1371/journal.pone.0201540)
Supplement: S2 Table — Effects of 21 days ENC® supplementation (20 mg/kg/day) for 21 days on liver phase I (A), phase II (B) and antioxidant (C) enzymes activities in RD and HFD rats. (DOCX) [file pone.0201540.s006.docx]

**Table S2 Effects of 21 days ENC^®^ supplementation (20 mg/kg/day) on liver phase I (A), phase II (B) and antioxidant (C) enzymes activities in RD and HFD rats.**

**(A)**

|  | **RD** | **RD + ENC^®^** | | | **HFD** | **HFD+ ENC^®^** | | |
| --- | --- | --- | --- | --- | --- | --- | --- | --- |
| **Day** | **0-21** | **7** | **14** | **21** | **0-21** | **7** | **14** | **21** |
| **NADPH-cyp red***^a^* | 6.66 ± 0.03 | 10.03 ± 0.99 ^##^ | 10.48 ± 0.69 ^##^ | 9.67 ± 1.01^##^ | 7.91 ± 1.10* | 12.01 ± 0.87 °° | 8.27 ± 0.70 | 10.66 ± 0.46 °° |
| **APD** (CYP3A1/2)*^a^* | 3.90 ± 0.12 | 7.90 ± 0.55 ^##^ | 6.68 ± 0.17 ^##^ | 5.78 ± 0.57^##^ | 7.53 ± 0.12** | 7.30 ± 0.46 | 6.96 ± 0.19 ° | 6.08 ± 0.64 °° |
| **p-NPH** (CYP2E1)*^a^* | 0.38 ± 0.06 | 0.39 ± 0.06 | 0.30 ± 0.05 | 0.37 ± 0.02 | 0.76 ± 0.04** | 0.43 ± 0.09 °° | 0.30 ± 0.02 °° | 0.19 ± 0.01 °°° ** |
| **ECOD***^a^* | 0.09 ± 0.01 | 0.13 ± 0.01 ^##^ | 0.13 ± 0.03 | 0.11 ± 0.03 | 0.10 ± 0.01 | 0.11 ± 0.01 | 0.09 ± 0.02 | 0.16 ± 0.01 °° |
| **PROD** (CYP2B1/2)*^b^* | 6.24 ± 0.10 | 6.32 ± 0.16 | 5.85 ± 0.33 ^##^ | 7.71 ± 0.24^##^ | 9.67 ± 0.14** | 10.31 ± 0.29° ** | 7.81 ± 0.23 °° * | 9.07 ± 0.41 * |
| **EROD** (CYP1A1)*^b^* | 10.29 ± 0.93 | 11.90 ± 1.14 | 7.97 ± 0.52 ^##^ | 9.20 ± 0.80 | 10.08 ± 1.05 | 8.11 ± 0.18° ** | 13.42 ± 0.65 °° ** | 5.94 ± 0.22 °° ** |
| **MROD** (CYP1A2)*^b^* | 10.16 ± 0.29 | 9.94 ± 0.38 | 8.82 ± 0.50 ^##^ | 10.05 ± 0.30 | 17.20 ± 1.53** | 12.20 ± 0.53 ** | 12.56 ± 0.35 ** | 13.11 ± 0.90 °° ** |

Data are Means Values ±S.D. *^a^*nmol×mg^-1^×min^-1^; *^b^*pmol×mg^-1^×min^-1^; RD0-21 or HFD0-21: pooled data between days 0-21. RD ENC^®^-treated groups *vs* RD0-21, ^##^*P*<0.01; HFD *vs* RD, same time, ^*^*P*<0.05, ^**^P<0.01; HFD-treated groups *vs* HFD0-21, °*P*<0.05, ^°°^P<0.01, °°°P<0.001.

**(B)**

|  | **RD** | **RD + ENC^®^** | | | **HFD** | **HFD+ ENC^®^** | | |
| --- | --- | --- | --- | --- | --- | --- | --- | --- |
| **Day** | **0-21** | **7** | **14** | **21** | **0-21** | **7** | **14** | **21** |
| **GST^a^** | 12.56 ± 1.04 | 13.75 ± 0.94 | 12.11 ± 0.94 | 22.37 ± 2.16^##^ | 11.57 ± 2.10 | 15.91 ± 0.96°° | 15.31 ± 0.34°° ** | 19.24 ± 0.35°° ** |
| **UDPGT^b^** | 2.61 ± 0.15 | 2.32 ± 0.39 | 2.17 ± 0.16 | 2.09 ± 0.4 | 1.48 ± 0.07** | 1.61 ± 0.19°° | 1.97 ± 0.11°° | 2.14 ± 0.16°° |

Data are Means Values ±S.D. ^a^μmol×mg^-1^×min^-1^. ^b^nmol×mg^-1^×min^-1^. RD0-21 or HFD0-21: pooled data between days 0-21. RD ENC^®^ treated groups *vs* RD0-21, ^##^*P*<0.01; HFD *vs* RD, same time, ^**^*P*<0.01; HFD treated groups *vs* HFD0-21, ^°°^P<0.01.

**(C)**

|  | **RD** | **RD + ENC^®^** | | | **HFD** | **HFD+ ENC^®^** | | |
| --- | --- | --- | --- | --- | --- | --- | --- | --- |
| **Day** | **0-21** | **7** | **14** | **21** | **0-21** | **7** | **14** | **21** |
| **CAT^a^** | 3.28 ± 0.44 | 4.79 ± 0.40^##^ | 4.74 ± 0.26^##^ | 4.42 ± 0.13^##^ | 3.6 ± 0.15 | 6.12 ± 0.45°° * | 2.81 ± 0.22°° ** | 8.43 ± 0.49°° |
| **NQO1^b^** | 4.54 ± 0.17 | 3.52 ± 0.22^##^ | 4.92 ± 0.60 | 3.76 ± 0.27^##^ | 4.83 ± 0.42 | 11.52 ± 1.15°° *** | 10.18 ± 0.86°° ** | 5.34 ± 1.14 |
| **SOD^b^** | 43.91 ± 2.73 | 45.88 ± 2.52 | 41.75 ± 0.93^##^ | 49.67 ± 1.60^##^ | 36.73 ± 2.12** | 48.00 ± 3.51°° | 33.29 ± 2.03° ** | 57.03 ± 5.48°° |
| **GSSG-red^a^** | 78.34 ± 5.15 | 80.35 ± 3.88 | 81.53 ± 6.90 | 86.55 ± 4.79 | 41.85 ± 0.91** | 74.45 ± 12.64°° | 77.24 ± 9.93°° | 116.21 ± 7.36°° |

Data are Means Values ±S.D.  ^a^μmol×mg^-1^×min^-1^; ^b^nmol×mg^-1^× min^-1^. RD0-21 or HFD0-21: pooled data between days 0-21. RD ENC^®^ treated groups vs RD0-21, ^##^*P*<0.01; HFD vs RD, same time, **P*<0.05, ^**^*P*<0.01; HFD treated groups vs HFD0-21, ^°^*P*<0.05, ^°°^*P*<0.01.
