# Supplementary material for: Liver and intestinal protective effects of Castanea sativa Mill. bark extract in high-fat diet rats
Source: PLoS One. 2018 Aug 6;13(8):e0201540. doi: 10.1371/journal.pone.0201540 (PMC6078294; doi:10.1371/journal.pone.0201540)
Supplement: S3 Table — Agonist [carbachol (CCh)] and antagonist (atropine) activities expressed as pEC50 or pA2, respectively, (A) and Calcium channels antagonist Nifedipine activity (B) in the isolated rat ileum and distal colon of RD- and HFD-rats supplemented with or without ENC® (20 mg/kg/day) for 21 days. (DOCX) [file pone.0201540.s007.docx]

**S3 Table. S3 Table. Agonist [carbachol (CCh)] and antagonist (atropine) activities expressed as pEC_50_ or pA_2,_ respectively, (A) and Calcium channels antagonist Nifedipine activity (B) in the isolated rat ileum and distal colon of RD- and HFD-rats supplemented with or without ENC^®^ (20 mg/kg/day) for 21 days.**

**(A)**

|  |  | **RD** | **RD + ENC^®^** | | | **HFD** | **HFD+ ENC^®^** | | |
| --- | --- | --- | --- | --- | --- | --- | --- | --- | --- |
|  |  | **0-21 days** | **7 days** | **14 days** | **21 days** | **0-21 days** | **7 days** | **14 days** | **21 days** |
| **Ileum** | **CCh*^a^*** | 6.27 ± 0.03 | 6.30 ± 0.05 | 6.28 ± 0.04 | 6.34 ± 0.05 | 5.89 ± 0.02 | 6.05 ± 0.03 | 6.01 ± 0.01 | 5.90 ± 0.04 |
|  | **Atropine*^b^*** | 8.95 ± 0.03 | 8.97 ± 0.01 | 8.88 ± 0.04 | 8.90 ± 0.05 | 8.68 ± 0.04 | 9.08 ± 0.02 | 8.26 ± 0.03 | 8.91 ± 0.02 |
| **Proximal colon** | **CCh*^a^*** | 5.92 ± 0.02 | 5.38 ± 0.06 | 5.40 ± 0.03 | 5.80 ± 0.02 | 5.83 ± 0.03 | 5.68 ± 0.01 | 5.57 ± 0.04 | 5.43 ± 0.02 |
|  | **Atropine*^b^*** | 8.77 ± 0.03 | 8.32 ± 0.06 | 8.45 ± 0.01 | 8.57 ± 0.04 | 7.73 ± 0.07 | 8.36 ± 0.04 | 8.27 ± 0.03 | 8.73 ± 0.02 |

*^a^*Data are expressed as pEC_50_. pEC_50_ = –log EC_50_. EC_50_ values are the means ± SE of at least four independent experiments and were calculated by a non-linear regression curve-fitting computer program [24]. *^b^*Data are expressed as p*A*_2_. p*A*_2_ values ± S.E. were calculated from Schild plots [25], constrained to slope -1.0 [26] (p*A*_2_ is the positive value of the intercept of the line derived by plotting log (DR – 1) *vs* log [antagonist]. The log (DR – 1) was calculated from three different antagonist concentrations, and each concentration was tested from four to six times. Dose-ratio (DR) values represent the ratio of the potency of the agonist carbachol (EC_50_) in the presence of the antagonist and in its absence. Parallelism of concentration–response curves was checked by linear regression, and slopes were tested for significance (P < 0.05).

**(B)**

|  |  | **RD** | **RD + ENC^®^** | | | **HFD** | **HFD+ ENC^®^** | | |
| --- | --- | --- | --- | --- | --- | --- | --- | --- | --- |
|  | **Days** | **0-21** | **7** | **14** | **21** | **0-21** | **7** | **14** | **21** |
| **Ileum** | **Activity*^a^*** | 88 ± 1.9 | 97 ± 0.7 | 95 ± 1.4 | 89 ± 2.3 | 98 ± 0.3*^d^* | 85 ± 2.2*^d^* | 89 ± 1.4 | 95 ± 2.6*^e^* |
|  | **EC_50_*^b^* (nM)** | 0.33 | 0.52 | 0.50 | 0.56 | 5.41 | 3.91 | 0.56 | 0.57 |
|  | **95% conf lim** | 0.25–0.50 | 0.26–0.60 | 0.35–0.59 | 0.49–0.61 | 4.83–6.34 | 1.83–5.04 | 0.48–0.61 | 0.18–0.59 |
| **Proximal colon** | **Activity*^a^*** | 80 ± 2.4*^c^* | 83 ± 1.6 | 88 ± 1.4 | 88 ± 2.6 | 51 ± 0.7 | 58 ± 2.5 | 84 ±2.6*^f^* | 93 ± 2.6*^c^* |
|  | **EC_50_*^b^* (nM)** | 0.25 | 0.30 | 0.71 | 1.01 | 9.43 | 6.57 | 2.20 | 0.72 |
|  | **95% conf lim** | 0.19–0.32 | 0.28–0.39 | 0.45–1.11 | 0.85–1.87 | 8.29–10.80 | 5.18–8.33 | 1.70–2.84 | 0.64–0.78 |

*^a^*Percent inhibition of Nifedipine in calcium-induced contraction on K^+^-depolarized (80 mM) guinea pig longitudinal smooth muscles, ileum and colon, at 10^-9^ M and 10^-8^ M respectively. The 10^-9^ M and 10^-8^ M concentrations gave the maximum effect in most tissues. RD0-21 or HFD0-21: pooled data between days 0-21. Activity reported as means ± S.E.M.. *^b^*Calculated from log concentration-response curves (Probit analysis by Litchfield and Wilcoxon, [26]) (with *n* = 6-7). When the maximum effect was <50%, the IC_50_ values were not calculated. *^c^*Attained at 10^-9^ M. *^d^*Attained ad 10^-8^ M. *^e^*Attained at 5x10
